# The Extracellular Matrix Promotes Diabetic Oral Wound Healing by Modulating the Microenvironment

**DOI:** 10.34133/bmr.0169

**Published:** 2025-03-19

**Authors:** Zhongke Wang, Li Wang, Sihan Wang, Hongmei Chen, Danni Wang, Aodi Li, Ying Huang, Yifan Pu, Xinlei Xiong, Xiangrui Lui, Yuwen Huang, Ling Guo

**Affiliations:** ^1^ Department of Prosthodontics, The Affiliated Stomatological Hospital of Southwest Medical University, Luzhou 646000, China.; ^2^ School of Stomatology, Southwest Medical University, Luzhou, Sichuan, China.; ^3^ Luzhou Key Laboratory of Oral & Maxillofacial Reconstruction and Regeneration, Luzhou 646000, China.

## Abstract

Oral wounds in diabetes mellitus (DM) often delay healing due to reduced angiogenesis and increased inflammatory response in the local microenvironment, even leading to graft necrosis and implant failure. Therefore, developing an effective program to promote healing is of great clinical value. Much of the current research is focused on promoting wound healing through surface adhesive materials that exert a pro-angiogenic, anti-inflammatory effect. However, the application of surface bonding materials in the oral cavity is very limited due to the humid and friction-prone environment. Decellularized extracellular adipose tissue (DAT) is an easily accessible and biocompatible material derived from adipose tissue. To further explore the potential of DAT, we used multi-omics to analyze its composition and possible mechanisms. Proteomic studies revealed that DAT contains anti-inflammatory, pro-angiogenic proteins that promote DM tissue regeneration. To adapt to the moist and chewing friction environment of the mouth, we modified DAT into a temperature-sensitive hydrogel material that can be injected intramucosally. DAT hydrogel has been verified to promote angiogenesis and exert anti-inflammatory effects through macrophage phenotypic transformation. Meanwhile, transcriptome analysis suggested that the inhibitory effect of DAT on the interleukin 17 signaling pathway might be a key factor in promoting DM oral wound healing. In conclusion, after multi-omic analysis, DAT hydrogel can exert good pro-angiogenic and anti-inflammatory effects through the interleukin 17 signaling pathway and can be adapted to the specific environment of the oral cavity. This provides a potential way to promote DM oral wound healing in a clinical setting.

## Introduction

Diabetes mellitus (DM) is a harmful chronic noncommunicable disease that affects 10% of the world’s adult population, estimated at 537 million people [1,2]. The high-glucose environment associated with DM leads to a variety of health complications, including cardiovascular disease, neuropathy, and ongoing trauma [3]. As the body’s largest organs, the skin and mucous membranes are the first lines of defense against any invasion that may disrupt the body’s homeostasis. However, DM often exacerbates local immune rejection [4,5] and vascular insufficiency [6,7], largely delaying the healing of this line of defense. Trauma healing is currently well characterized and treated in cutaneous trauma, but the moist, friction-prone environment of the mouth reduces the clinical translation of treatment options. It has been reported that more than 30% of adults suffer from oral wounds due to trauma, implants, and tumor resections, which severely impact the quality of life of patients [8]. Under normal circumstances, oral wounds usually heal faster and leave less visible scars than skin wounds because inflammation in oral mucosal wounds is more transient than in the skin [9,10]. However, the rate of wound healing can be markedly inhibited in specific cases, especially when suffering from DM. In severe cases, this can lead to necrosis of the graft flap [11] and dental implant failure [12]. Therefore, finding material that promotes the healing of DM oral wounds is essential.

Currently, autografts of localized tissue are the treatment of choice for filling marked oral defects, and area-limited wounds are often closed using only suturing. However, local wound healing is usually slow, and even graft failure occurs due to increased microenvironmental vascular insufficiency and inflammatory response in the wound due to DM [13,14]. In recent years, therapies used to promote the healing of oral wounds have focused on delivering cells via scaffolds adhering to the wound surface, exosomes [15] to promote tissue regeneration, or immunomodulators such as doxycycline [16] or ginsenoside RG1 [17] to exert anti-inflammatory effects. Among these scaffolds, good efficacy has been achieved by material synthesis mimicking the extracellular matrix (ECM) [18]. These scaffolds have components or structures similar to the ECM, thus favoring cell attachment and promoting wound healing. Therefore, the ideal materials to promote DM oral wound healing should have anti-inflammatory, excellent biocompatibility, and angiogenesis-promoting properties while adapting to the moist environment of the oral cavity.

A decellularized extracellular matrix (dECM) is a functional biomaterial usually prepared by removing immunogenic cellular components from mammalian tissues/organs via decellularization techniques [19]. A dECM retains the original physicochemical signatures and biological properties of the ECM and can provide 3-dimensional scaffolds and growth environments for cells [20]. dECMs have demonstrated outstanding potential for various biomedical applications and clinical translations. So far, they have been widely used in regenerative medicine for repairing tissues and organs [21–23]. In recent years, dECM-derived hydrogels have also been developed [24]. These hydrogels retain the biocompatibility of dECMs and have temperature-controlled properties, which allow them to be converted from liquid to hydrogel at near-body temperature (37 °C) [25]. This injectable type brings more clinical application scenarios for dECMs. Decellularized extracellular adipose tissue (DAT) is one of the many dECMs that are widely used for the regeneration of a variety of tissues, such as adipose [26,27], bone [28], and vascular tissues [29]. DAT has been reported to have a role in promoting endothelial cell migration and facilitating macrophage polarization toward M2 [30]. However, none of the above studies reported the underlying mechanism. In contrast, the introduction of multi-omics has helped us discover new application areas for DAT and further explore its mechanism of action. To the best of our knowledge, few studies have utilized DAT to promote DM oral wound healing. On the one hand, DAT possesses conditions that promote DM wound healing, such as promoting neoangiogenesis and macrophage transformation to the M2 type. On the other hand, DAT hydrogels can be used for submucosal injection gelation through their injectable, temperature-sensitive properties, avoiding the wet, friction-prone environment of the oral cavity.

**Fig. 1.**
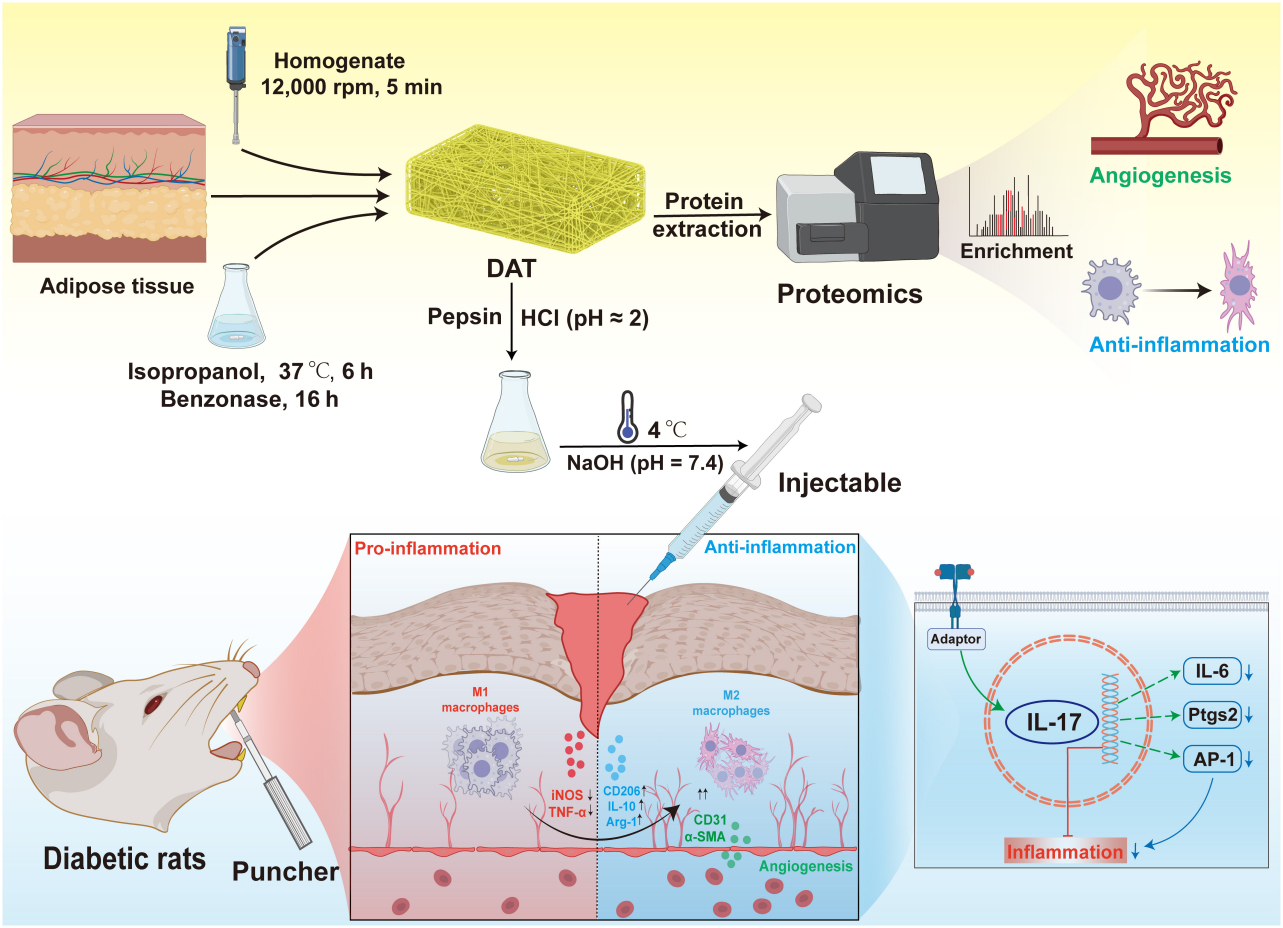
Fabrication of decellularized extracellular adipose tissue (DAT) hydrogel and its promotional effect on diabetic oral wound healing with schematic mechanism. iNOS, inducible nitric oxide synthase; TNF-α, tumor necrosis factor alpha; IL-10, interleukin 10; Arg-1, arginase 1; α-SMA, alpha smooth muscle actin; IL-17, interleukin 17; IL-6, interleukin 6; Ptgs2, prostaglandin-endoperoxide synthase 2; AP-1, activator protein 1.

In this study, we innovatively used a multi-omic approach to investigate the promotional effects and possible mechanisms of DAT hydrogel on DM oral wound healing (Fig. 1). Firstly, the extracted DAT was analyzed by shotgun proteomics and found to have the potential to promote angiogenesis, anti-inflammation, and other properties for DM wound healing. The results of cellular experiments showed that DAT hydrogel promoted endothelial cell proliferation, migration, and vasculogenesis. Meanwhile, the anti-inflammatory and antioxidant effects of DAT hydrogel were also obvious. After treatment of palatal defects in DM rats with DAT hydrogel, it was found that the wound healing time was shortened, the rate of neovascularization was increased, and M1 macrophages decreased while M2-type macrophages increased. Finally, the mechanism by which DAT promotes the healing of DM oral tissues was further explained by transcriptomics.

## Materials and Methods

### Porcine adipose decellularization and hydrogel production

The adipose tissue decellularization process is modified from classic protocols [31,32]. Porcine subcutaneous adipose tissue was collected from a local slaughterhouse and transported on ice to the lab. Once in the lab, the adipose tissue was cut into small pieces and kept on ice the whole time. DAT was obtained by homogenization (Shanghai specimen model factory, China) (12,000 rpm, 5 min), centrifugation in ultrapure water (5,000 rpm, 5 min), and manual discarding of lipids (retention of the precipitate). After washing with phosphate-buffered saline (PBS; Solarbio, China), the residue was subjected to polar solvent extraction in 99.9% isopropanol (Macklin, China) for 48 h to remove the lipid components. Afterward, the samples were incubated for 16 h with Benzonase (Novoprotein, China) digestion solution. After 3 washes with PBS, the samples were sterilized by rinsing with 75% alcohol and subsequently lyophilized (Scientz, China) until completely dry. To facilitate the production of hydrogels later, all lyophilized DATs were ground into powder using a grinder and subsequently sealed to isolate moisture and stored in a refrigerator at 4 °C.

The DAT was digested with 2 mg/ml pepsin (Macklin, China) in 0.01 M HCl (Macklin, China) for 48 h with agitation. After complete digestion, the pH was adjusted to 7.4 using 0.1 M NaOH (Macklin, China) at 4 °C, and the osmolality was adjusted with 10× PBS (Solarbio, China). The above-synthesized pre-DAT hydrogels were stored at 4 °C to prevent them from gelating [33]. For use, the pre-gel was incubated at 37 °C for 15 min to form a gel [34].

Adding different volumes of PBS during the equilibration of osmotic pressure can yield different concentrations of pre-DAT hydrogels. Pre-experiments on gel formation and injectability were performed on pre-DAT hydrogels with different concentrations.

### Characterization of decellularization

#### DNA and collagen quantification

DNA from DAT and fresh subcutaneous adipose tissue was extracted and quantified to test decellularization efficacy. DNA was extracted using Rapid Animal Genomic DNA Isolation Kit (Sangon Biotech, China). Subsequently, the extracted DNA was quantified by a NanoDrop microspectrophotometer (Thermo Fisher Scientific, USA).

A total protein assay kit (Yanaye, China) extracted total collagen from DAT and fresh adipose tissue. Collagen was quantified at 450 nm on a microplate reader (Synergy H1, BioTek, USA).

#### Histological analysis

Fresh porcine subcutaneous adipose tissue and DAT were fixed, embedded in paraffin, and cut into 5-μm sections. To evaluate decellularization efficacy, hematoxylin–eosin (HE) staining (Servicebio, China), Masson staining (Servicebio, China), Oil Red O staining (Servicebio, China), and 4′,6-diamidino-2-phenylindole (DAPI) cytosolic staining (Servicebio, China) were used. Photographs of sections were captured with a slide scanner (Leica, Wetzlar, Germany) and an inverted fluorescence microscope (Olympus, Japan).

### DAT hydrogel characterization

#### Fourier transform infrared spectroscopy

Fourier transform infrared spectroscopy (Beifen-Ruili, China) was used to analyze the chemical structures of DAT and DAT hydrogels. The spectral range was configured as 400 to 4,000 cm^−1^, and the mode was set to transmittance. The data were imported into Origin for analysis.

#### Surface morphology characterization

Varying concentrations of pre-DAT hydrogels were placed in a 24-well plate and incubated at 37 °C for 15 min to fully gelate. The samples were then prepared by freezing at −20 °C for 24 h, followed by freeze-drying in a vacuum freeze dryer (Labconco, USA) for 18 h. Samples are frozen in liquid nitrogen for 2 min before testing. Sections were cut with a scalpel blade to reveal cross-sections. The cross-sections of all samples were subsequently sprayed with gold. The cross-sectional morphology of the samples was observed using 3 random images captured by a scanning electron microscope (Inspect F50, Thermo Fisher, USA). Images were imported into ImageJ to calculate the porosities and compare them for different DAT concentrations (*n* = 3).

#### Rheological properties

The rheological properties of the DAT hydrogels with increasing temperature were analyzed using a rheometer (TA, USA). The pre-DAT hydrogels were placed on a parallel plate at 4 °C. The temperature of the parallel plates was increased from 4 to 37 °C at a rate of 2 °C/min. The storage modulus (*G*′) and loss modulus (*G*″) were monitored in oscillatory time-scan mode at a strain of ∼1% and a frequency of ∼1 Hz.

### Shotgun proteomic analysis and data analysis

Briefly, the proteins were digested by trypsin and the digested peptides of DAT were desalted on a C18 column, concentrated by vacuum centrifugation and subsequently reconstituted in formic acid. The data were then exported for protein quantification and identification. The identified proteins were subjected to cluster analysis, subcellular localization, domain annotation, Gene Ontology (GO) annotation, Kyoto Encyclopedia of Genes and Genomes (KEGG) annotation, enrichment analysis, and protein–protein interaction analysis.

### Hemolytic potential of DAT hydrogels

A hemolysis test was performed based on previous studies [35]. Red blood cells were obtained by collecting fresh blood from New Zealand rabbits. The red blood cells were then resuspended in PBS at a volume concentration of 5% (v/v). The erythrocyte suspensions were then incubated with DAT hydrogels (4, 6, and 8 mg/ml) for 1 h at 37 °C. Sterile PBS and 0.1% Triton X-100 were negative and positive controls, respectively. To obtain the supernatant, all samples were centrifuged. Then, 100 μl of the supernatant from each sample was transferred to a 96-well plate. The absorbance was measured at 540 nm using an enzyme marker (Synergy H1, USA). The hemolysis rate was calculated as (absorbency(test) − absorbency(negative))/(absorbency(positive) − absorbency(negative)) ∗ 100%. Finally, the erythrocytes were mixed and placed in 24-well plates. The erythrocytes were observed under a microscope (Lecia, Germany) and photographed for records.

### Cell culture

To verify DAT’s biocompatibility, angiogenesis-promoting, and anti-inflammatory properties, mouse macrophage RAW264.7 and human umbilical vein endothelial cells (HUVECs) were purchased and passaged (Procell, China). Both types of cells were cultured in a highly glycosylated Dulbecco’s modified Eagle medium (Gibco, USA) containing 10% fetal bovine serum (Procell, China) and 1% streptomycin/penicillin (Beyotime, China). The culture environment was set at 37 °C and 5% CO_2_. Cells were passaged when they grew to 85% to 90%, and passages were digested with trypsin (Gibco, USA). Subsequent experiments were performed using 2 to 5 generations of cells.

### Cell viability and proliferation

#### Live/dead assay

The varying concentrations of DAT hydrogels were lyophilized and ultraviolet sterilized for 30 min. Then, they were immersed in Dulbecco’s modified Eagle medium complete medium at 4 °C. After 48 h of immersion, they were filtered through a 0.22-μm filter (Jet Biofil, China). Subsequent live/dead cell staining assays were performed, and both types of cells were cultured in 24-well plates. After the cells had adhered to the plates, they were treated for 1, 3, and 5 d using extracts from different samples. The plates were washed 3 times with PBS. PBS containing live/dead stain (US Everbright, Suzhou, China) was added, incubated at 37 °C and 5% CO_2_, and protected from light. The cells were observed under an inverted fluorescence microscope (Leica, Wetzlar, Germany) after 20 min of incubation.

#### Cell Counting Kit-8

The Cell Counting Kit-8 (CCK-8) assay was used to determine cell viability. In brief, the cells were cultured in 96-well plates at a density of 5,000 cells per well. After the cells had adhered to the plates, they were treated for 1, 3, and 5 d using extracts from different samples. They were washed 3 times using PBS and 100 μl of medium (containing 10% CCK-8 [APExBIO, USA]) per well according to the CCK-8 instructions. They were then incubated at 37 °C and 5% CO_2_ for 2 h and measured using a microplate reader (Synergy H1, BioTek, USA).

### In vitro angiogenesis assay experiments

#### Wound healing assay

The role of the DAT hydrogel in promoting HUVEC migration was assessed using the wound healing assay; 2 to 5 generations of HUVECs were inoculated in 6-well plates at a density of 2.5 × 10^5^ per well, and cells were cultured overnight. The medium was discarded when the density reached 90%, and monolayers of cells were scraped using a 200-μl sterile pipette tip. The cells were then treated for 12, 24, and 36 h according to the protocol in the “Wound healing assay” section and photographed at the appropriate time points. The average percentage of area migrated compared to the original wound area was calculated to assess the rate of cell migration.

#### Transwell assay

For migration capacity analysis, HUVECs were seeded in the upper layer of Transwell cell culture chambers. In the upper layer of the Transwell culture chamber, 50 μl of serum-free medium was added. A control (complete medium containing 10% fetal bovine serum) was placed below the cell-permeable membrane, and an immersion solution was used with a concentration of DAT hydrogel. After 24 h of incubation, the upper layer of nonmigrated HUVECs was removed, and the HUVECs that had already migrated was fixed with 4% paraformaldehyde (PFA) for 30 min. Staining was performed using a crystal violet staining solution for 30 min away from light. The observation was performed using a light microscope (SOPTOP, China), and 3 random fields of view per well were photographed and the cells were counted.

#### Tube formation assay

Tube formation assays were used to assess the ability of DAT hydrogels to promote blood vessel formation. HUVECs were pretreated for 24 h using the protocol in the “Live/dead assay” section using different concentrations of DAT hydrogels; 48-well plates were pretreated using Matrigel (Corning, USA), spread overnight at 4 °C, and cured for 2 h at 37 °C. Pretreated HUVECs were inoculated at 5 × 10^4^ per well and incubated at 37 °C with 5% CO_2_. Photographs were taken at 4 and 6 h of incubation, and the pictures were imported into ImageJ to analyze the quantification of tubular networks (including the number of master junctions, number of nodes, and total segment length).

#### Immunostaining

For immunostaining experiments, we inoculated 1 × 10^4^ HUVECs into 24-well plates. Subsequently, they were divided into a blank control group and groups with different concentrations of DAT hydrogel extracts. Cells were rinsed with PBS, fixed in 4% PFA for 15 min. and permeabilized with 0.1% Triton X-100 after 24 h. Then, the samples were blocked with 5% goat serum (Beyotime, China) for 1 h at room temperature. Primary antibodies were incubated with samples overnight at 4 °C: CD31 (1:300, Servicebio, China) and alpha smooth muscle actin (α-SMA) (1:1,000, Servicebio, China). Then, a goat anti-rabbit immunoglobulin G secondary antibody (Alexa Fluor 594) (1:100, Servicebio, China) was incubated with the corresponding samples for 1 h at room temperature. Finally, the nuclei of HUVECs were stained with DAPI (Servicebio, China). The crawls were removed and placed on slides with an antifluorescence quencher. Fluorescence images were captured using an inverted fluorescence microscope.

### In vitro anti-inflammatory and antioxidant assays

#### Anti-inflammatory-related gene expression

To simulate the inflammatory response to the wound healing process in vitro, RAW264.7 was inoculated into a 6-well plate and treated with 500 ng/ml lipopolysaccharide (LPS) (Solarbio, China) for 24 h. This was followed by incubation with DAT hydrogel extract for 48 h. All samples were processed with a TRIzol RNA extraction kit (Invitrogen, Carlsbad, CA, 15596-026) in accordance with the total RNA extraction procedure. A NanoDrop spectrophotometer (Thermo Fisher Scientific, USA) was used to quantify RNA by measuring absorbance at 260 nm. The RNA was then reverse-transcribed to complementary DNA, and real-time reverse transcription polymerase chain reaction (RT-qPCR) was performed to assess the expression of anti-inflammatory-related genes (*iNOS*, *Cd206*, *TNF-α*, *Il10*, and *Arg-1*) using SYBR FAST qPCR Master Mix (Kapa Biosystems, USA). The primer sequences are shown in Table S1.

#### Immunofluorescence of the anti-inflammatory properties of DAT hydrogels

RAW264.7 was inoculated into the crawling slices of 24-well plates and treated as the blank control group, LPS group, and LPS + different concentrations of DAT hydrogel extract group. Cells were washed with PBS, fixed in 4% PFA for 15 min, and permeabilized with 0.1% Triton X-100 after 24 h. The samples were then closed with 5% goat serum (Beyotime, China) and left at room temperature for 1 h. The following primary antibodies were incubated with the samples overnight at 4 °C: CD206 (1:200, Proteintech, USA) and inducible nitric oxide synthase (iNOS) (1:200, Proteintech, USA). A secondary goat anti-rabbit immunoglobulin G antibody (Alexa Fluor 594) (1:100, ZSGB-BIO, China) was then incubated with the corresponding samples for 1 h at room temperature. Finally, the cytoskeleton and nucleus of RAW264.7 were stained with DAPI (Solarbio, China). Crawls were removed and placed on a slide sealed with a drop of the antifluorescence quencher. Fluorescence images were captured using an inverted fluorescence microscope (Olympus, Japan).

#### Antioxidant determination of DAT hydrogels

In brief, RAW264.7 cells were inoculated into 24-well plates, and reactive oxygen species (ROS) were induced with hydrogen peroxide for a period of 6 h. The H_2_O_2_ solution for the experimental group was configured using different concentrations of DAT hydrogel infusion. Then, intracellular ROS levels were characterized using 2,7-dichlorodihydrofluorescein diacetate (DCFH-DA) (Solarbio, China), while the nuclei were stained with Hoechst. At the same time, we performed live/dead staining of different groups of cells using the method in the “Transwell assay” section. Subsequently, observation and photographic documentation were conducted using a fluorescence microscope.

### Animal experiments

#### In vivo compatibility test

In in vitro experiments, we used 3 concentrations of DAT hydrogels in exactly the same soaking solution medium and concluded that the 6 mg/ml group had better anti-inflammatory and angiogenic effects. We hypothesized the reason for this may be that the gap size of the hydrogel affects the release of the active substance. On the premise that it was sufficient to conclude that DAT hydrogel promotes diabetic oral wound healing, we chose to use 6 mg/ml DAT for subsequent in vitro experiments to be more animal ethical.

Male Sprague Dawley (SD) rats (6 to 8 weeks old, 190 to 210 g) were obtained at the beginning of the study and subsequently placed in specific-pathogen-free conditions to complete the experiments. All animal experiments in this study were approved by the Experimental Animal Ethics Committee of Southwest Medical University (SWMU20240019). To validate the compatibility and in vivo degradability of DAT hydrogels, we injected DAT hydrogel subcutaneously into the rats for experiments. The control group was injected with an equal amount of saline. After 2 weeks of injection, local subcutaneous tissues and viscera were taken for HE staining.

#### Establishment of a palatal wound model in DM rats

After a week of acclimatization of 6 to 8 weeks of SD rats in the environment, the experimental group was fed high-fat chow containing 40% fat. After 3 weeks of feeding, they were fasted for 12 h and injected with a small dose of streptozotocin (35 mg/kg). After 3 d of injection, the blood glucose values of rats in the modeling group exceeded 250 mg/dl 6 h after feeding. Three days after the injection, blood glucose was measured, and rats with blood glucose higher than 250 mg/dl were considered to have successful DM modeling.

The above DM rats were randomly divided into DM and DAT groups, while rats with normal blood glucose and the same number of weeks were used as a blank control group. The rats were anesthetized by intraperitoneal administration of sodium pentobarbital (30 mg/kg). A soft-tissue defect straight to the bony surface was prepared using a gingival ring knife in the middle of the rat palate. The defect was approximately 2 mm in diameter and 0.5 mm in depth. The mucoperiosteal flap was then excised to create a circular defect. The DAT group was injected into the soft tissue around the palatal incision using DAT hydrogel. Meanwhile, the DM and control groups were injected with saline. Injections were given every 24 h. An overdose of sodium pentobarbital (80 mg/kg body weight) was injected on 0, 3, 7, and 14 d to execute the wound and to take wound maps.

#### Histological analysis of palatal wound healing in DM rats

Animal samples from the “Establishment of a palatal wound model in DM rats” section were removed and fixed in PFA at 4 °C and demineralized in a 10% EDTA solution at room temperature. Following the complete demineralization process, the specimens were subjected to a dehydration phase, embedded in paraffin wax, and subsequently sectioned at a thickness of 4 μm. According to the manufacturer’s instructions, each specimen was stained with HE histological stain (Servicebio, Wuhan, China), Masson trichrome (Servicebio, Wuhan, China), and immunohistochemical staining (tumor necrosis factor alpha [TNF-α] and interleukin 1β [IL-1β], Servicebio, Wuhan, China). Micrographs were taken after the above histological staining using a section scanner (Leica, Wetzlar, Germany).

#### Immunofluorescence of DAT hydrogels promoting angiogenesis and anti-inflammation in vitro

In order to conduct immunofluorescence staining, the dewaxed slides were immersed in citrate–EDTA antigen repair solution (Servicebio, China) for a 15-min period at a temperature of 95 °C. Following this, the solution was cooled to room temperature and allowed to sit for 1 h. Sections were permeabilized with 0.1% Triton X-100 (Solarbio, Beijing, China) for 10 min and closed in 5% bovine serum albumin (Servicebio, China) for 1 h at room temperature. Then, they were incubated with primary antibodies against CD206 (1:4,000, Proteintech, USA), iNOS (1:2,000, Proteintech, USA), CD31 (1:100, Servicebio, China), and α-SMA (1:200, Servicebio, China) at 4 °C overnight. After incubation, the specimens were washed 3 times with PBS. Using the secondary antibody, they were incubated for 1 h at room temperature. Nuclei were stained with DAPI (Servicebio, China). Immunofluorescence images were obtained using a confocal microscope (FV3000, Olympus, Tokyo, Japan). The fluorescence intensity was subsequently analyzed using the ImageJ software.

#### RNA-seq and data analysis

The palatal defect model of DM rats was established as described above. They were then randomized into 2 groups: (a) the DM group, injected with saline, and (b) the drug group, injected with DAT hydrogel. Palatal tissues were taken for RNA sequencing (RNA-seq) after 7 d of treatment. Total RNA was extracted from soft tissues of the rat palate using TRIzol reagent (Magen). The RNA samples’ *A*_260_/*A*_280_ absorbance ratio was measured using NanoDrop ND-2000 (Thermo Scientific, USA). The RNA integrity number of the RNA was determined using Agilent Bioanalyzer 4150 (Agilent Technologies, CA, USA). Only RNAs that had been deemed to meet the requisite quality control standards were used for library construction. The preparation of paired-end libraries was conducted in accordance with the instructions provided by ABclonal mRNA-seq Lib Prep Kit (ABclonal, China). Sequencing was performed with the Illumina NovaSeq 6000/MGISEQ-T7 sequencing platform. Raw reads in fastq format are processed using a Perl script to remove low-quality reads and obtain clean reads for subsequent analysis. Clean reads were then mapped to the reference genome using HISAT2. The number of fragments per kilobase per million was calculated for each gene. Differential expression analysis of genes between groups was performed using DESeq2 with default screening thresholds for differentially expressed genes of |log_2_FC| > 1 and *P*.adj < 0.05. GO and KEGG enrichment analyses were performed on the differential genes. Function enrichment and KEGG pathway enrichment analyses were performed using the clusterProfiler R package, and when *P* < 0.05, this GO or KEGG function was considered significantly enriched.

#### Initial validation of anti-inflammatory pathways

To further validate RNA-seq, we performed experiments using rats treated according to the “Establishment of a palatal wound model in DM rats” section for 7 d. RNA from the samples was extracted as per the “Anti-inflammatory-related gene expression” section, and RT-qPCR was performed to assess the expression of interleukin 17 (IL-17) signaling pathway-related genes (*Il17*, *Il6*, *Ap-1*, and *Ptgs2*). Primer sequences are shown in Table S2.

Rats treated for 7 d in the DM and DAT groups (“Establishment of a palatal wound model in DM rats” section) were removed for experiments. Total proteins were extracted from the radioimmunoprecipitation assay buffer in which the developing soft tissues of the rat palate were removed and placed. The total protein concentration was determined in accordance with the instructions provided with the bicinchoninic acid kit (Beyotime, China). Subsequently, equal quantities of protein were subjected to sodium dodecyl sulfate–polyacrylamide gel electrophoresis and transferred onto polyvinylidene fluoride membranes (Millipore, USA). After being closed with 5% skimmed milk for 1 h, the membranes were probed with the following primary antibodies overnight at 4 °C: activator protein 1 (AP-1; 1:1,000, Bioswamp, China), interleukin 6 (IL-6; 1:1,000, Bioswamp, China), prostaglandin-endoperoxide synthase 2 (PTGS2; 1:1,000, Bioswamp, China), IL-17 (1:1,000, Bioswamp, China), and glyceraldehyde-3-phosphate dehydrogenase (1:2,000, Bioswamp, China). After washing with Tris-HCl-buffered saline and Tween (TBST; Beyotime, China, ST673), the membranes were incubated with a secondary antibody (horseradish peroxidase labeled, 1:5,000, Beyotime, China, P0023) for 1 h at room temperature. After washing again with TBST, the mixed enhanced chemiluminescence luminescent solution was added to the front side of the membrane. Protein signals were detected using a fully automated chemiluminescence analyzer (Tanon-5200, China), and protein quantification was performed using the ImageJ software.

### Statistical analysis

All quantitative data are expressed as mean ± standard deviation. Data were analyzed by *t* test (2 groups) or analysis of variance (ANOVA; multiple groups) which depends on the number of groups, followed by Tukey’s multiple-comparisons test (GraphPad Prism 10.0 software, USA). Differences were considered not statistically significant (ns) when *P* > 0.05 and statistically significant when *P < 0.05, **P < 0.01, ***P < 0.001, and ****P < 0.0001.

## Results

### Extraction of DAT from porcine adipose tissue and synthesis of hydrogels

**Fig. 2.**
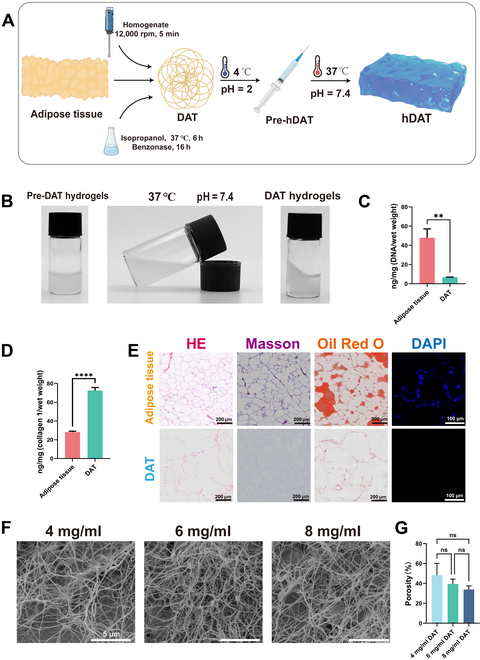
(A) Fabrication process of DAT hydrogels. (B) Comparison of DAT hydrogels before and after gel formation. (C) DNA content of adipose tissue and DAT. (D) The collagen content of adipose tissue and DAT. (E) Comparison plots of adipose tissue and DAT subjected to hematoxylin–eosin (HE) staining, Masson staining, Oil Red O staining (scale bar: 200 μm), and 4′,6-diamidino-2-phenylindole (DAPI) staining (scale bar: 50 μm). (F) Transmission electron microscopy (TEM) images of DAT hydrogels with different concentrations (scale bar: 5 μm). (G) TEM image porosity difference plots of DAT hydrogels with different concentrations. Statistical difference expression: ns, not significant; *P* > 0.05; **P < 0.01; ****P < 0.0001; analysis was performed using a *t* test with one-way analysis of variance (ANOVA), *n* = 3. hDAT, DAT hydrogel.

Subcutaneous adipose tissue was decellularized to white fibrous-like tissue by the protocol in the “Porcine adipose decellularization and hydrogel production” section (Fig. 2A). To avoid the oral cavity’s moist, friction-prone environment, we propose administering the drug by submucosal injection. The obtained DAT was lyophilized and ground into powder. DAT can be enzymatically cleaved to an injectable state and subsequently re-cross-linked chemically and physically to form a gel at a specific temperature (37 °C) and a specific pH (7.4). Subsequently, we prepared DAT hydrogels of 2, 4, 6, 8, and 10 mg/ml for submucosal injection. However, the 10 mg/ml hydrogel could not be injected through a syringe, and the 2 mg/ml hydrogel was found to be difficult to form into a gel. Therefore, we chose 4, 6, and 8 mg/ml for our subsequent experiments.

We then observed the temperature sensitivity of the DAT hydrogel. As shown in Fig. 2B, the DAT pre-hydrogel will form a gel when the pH is adjusted to 7.4 and the temperature reaches 37 °C. This suggests that DAT hydrogels have the potential to form hydrogels after injection into the submucosal layer. This provides us with a basis for designing intramucosal injectable hydrogels that avoid oral friction.

By comparing the DNA content with that of natural porcine subcutaneous adipose tissue (95.77 ± 9.32 ng/mg), we found that the DNA content (1.65 ± 0.01 ng/mg) in DAT was reduced by more than 95% (Fig. 2C). The double-stranded DNA value was lower than the internationally recognized standard of 50 ng/mg [36], indicating successful decellularization. Meanwhile, quantitative analysis of collagen showed that the collagen in the adipose tissue was well preserved after decellularization (Fig. 2D). The removal of nuclei was further determined by HE staining and DAPI staining. The adipose tissue removal and fibronectin retention were determined by Masson and Oil Red O staining (Fig. 2E). The above results indicated that we successfully completed the decellularization procedure, removing most of the immunogenic cellular components.

Since DAT mainly comprises collagen fibers, pepsin digestion is necessary for making hydrogels. Fourier transform infrared spectroscopy was used to detect whether pepsin digestion harms the main components of DAT (Fig. S1). The gel-forming process of DAT hydrogels was not observed to have a significant impact in the central peak (amide A [3,440 to 3,300 cm^−1^], amide B [2,990 to 2,830 cm^−1^], amide I [1,640 cm^−1^], amide II [1,550 cm^−1^], and amide III [1,240 cm^−1^]). The above results show that the structure of DAT did not have significant changes during hydrogel formation.

The scanning electron microscope results of DAT hydrogel materials with different concentrations (Fig. 2F) show that the interior of the hydrogel consists of a reticulated structure and many fiber-like filaments. The differences found by statistical analysis of the porosity of hydrogels with different concentrations were not statistically significant (Fig. 2G).

The rheological analysis demonstrated that the storage modulus (*G*′) and loss modulus (*G*″) of the DAT hydrogel solutions exhibited a rapid increase and reached a stable plateau following the warming of the solutions to 37 °C. This phenomenon is indicative of the formation of hydrogels (Fig. S2A to C). There was little difference in the time required for the 3 different concentrations of DAT hydrogels to reach the stabilization plateau (1,400 s). The various concentrations of hydrogels exhibited solid-state behavior at 37 °C, which allows DAT hydrogels to be used as an injectable biomaterial for filling tissue defects.

### Shotgun proteomic analysis reveals the potential tissue growth-promoting capacity and pathways of DAT

**Fig. 3.**
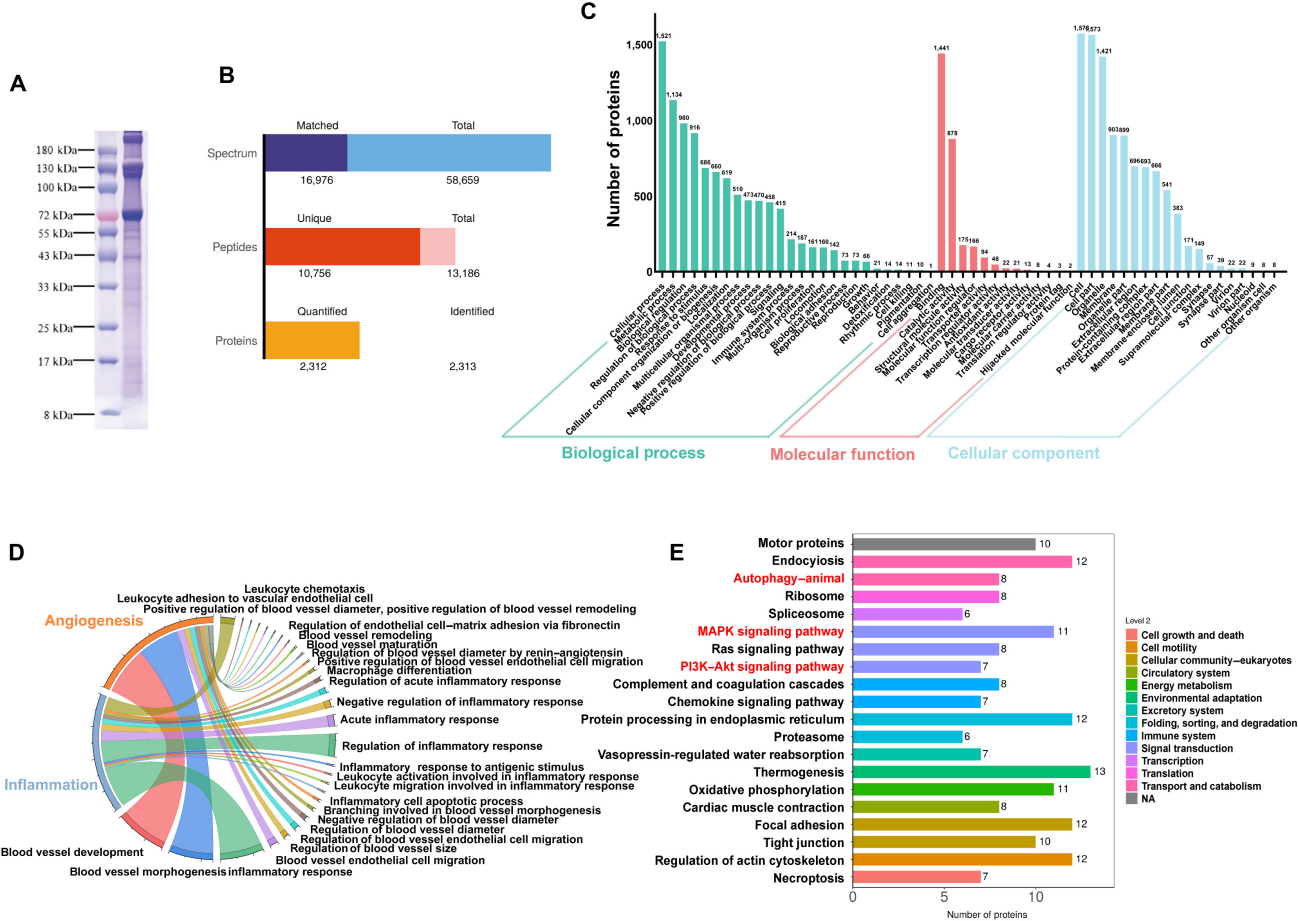
(A) Proteins and peptides were purified by gel electrophoresis and analyzed for approximate distribution. (B) The number of protein peptides that were extracted, analyzed, and quantified. (C) DAT’s top 30 Gene Ontology (GO)-enriched terms. (D) Chordal graph composed of GO terms related to angiogenesis and inflammation. (E) Kyoto Encyclopedia of Genes and Genomes (KEGG) bioprocess enrichment analysis of DAT. MAPK, mitogen-activated protein kinase; PI3K, phosphoinositide 3-kinase.

We performed a shotgun proteomic analysis to gain an in-depth understanding of DAT composition. Figure 3A shows the gel electrophoresis of DAT after protein extraction, which showed clear bands and peptides concentrated around 72, 130, and 180 kDa. A total of 13,186 peptides were identified, and 2,312 were quantitatively analyzed (Fig. 3B). We further analyzed the functional bioinformatics of DATs to identify the functional pathways in which they are involved (Table S3). To ascertain the role of DAT in biological processes, cellular components, and molecular functions, a subsequent GO analysis was conducted (Fig. 3C). The results showed that DAT was involved in the development process, immune system process, cell proliferation, and growth. To further understand whether DAT can promote diabetic oral wound healing, we mapped angiogenesis- and anti-inflammatory-related chordal diagrams (Fig. 3D). The KEGG pathways were analyzed (Fig. 3E), and multiple inflammation-related pathways were identified from the first 30: autophagy, mitogen-activated protein kinase signaling pathway, and phosphoinositide 3-kinase–Akt signaling pathway. The results of KEGG provide theoretical support for further exploration of the mechanism of action of DAT. These findings show that DAT correlates with tissue growth, angiogenesis, and inflammation. Accordingly, we hypothesize that DAT can be applied to DM oral wound healing by controlling inflammation and promoting tissue growth.

### Cytocompatibility and proliferation of DAT hydrogels

**Fig. 4.**
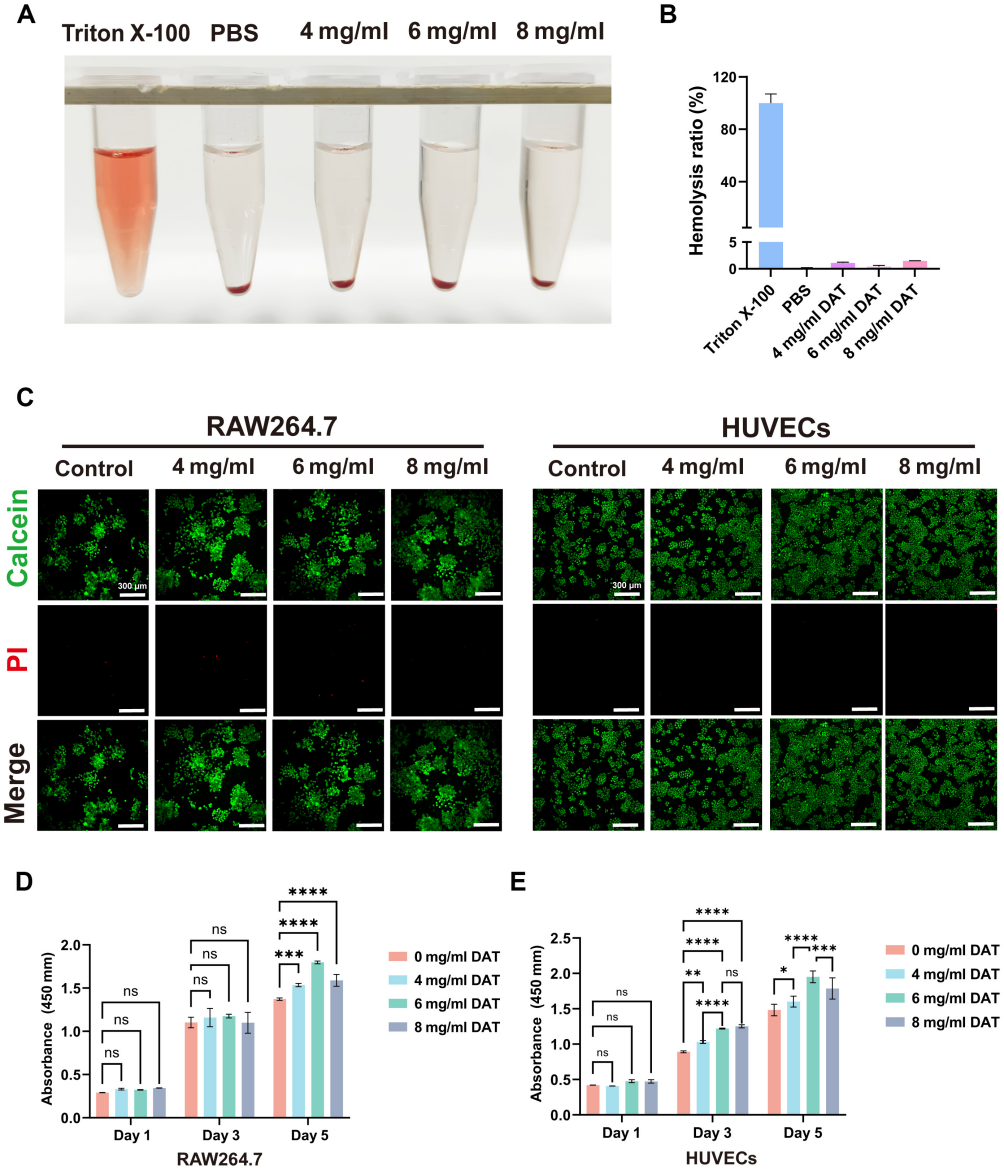
(A) Different hydrogel concentrations affect rabbit erythrocyte hemolysis, with phosphate-buffered saline (PBS) as the negative control and Triton X-100 as the positive control. (B and C) Cell Counting Kit-8 (CCK-8) assay to assess the cytotoxic/proliferative effects after 1, 3, and 5 d of co-incubation with different hydrogels (macrophages (B) and endothelial cells (C)). (D and E) Live/dead staining to assess the toxic effect of hydrogels (scale bar = 300 μm) (macrophages (D) and endothelial cells (E)). Statistical difference expression: ns, *P* > 0.05; *P < 0.05; **P < 0.01; ***P < 0.001; ****P < 0.0001; analyses were performed using ANOVA, *n* = 3. HUVECs, human umbilical vein endothelial cells; PI, propidium iodide.

Fresh erythrocytes were treated with different concentrations of DAT hydrogel to determine its cytotoxicity. The results indicated that the positive control group showed a distinct red color, and broken erythrocytes were also visible under the microscope (Fig. S3). The hemolysis rate of the experimental groups with different concentrations (4, 6, and 8 mg/ml) was less than 3% (Fig. 4A). In contrast, the microscopic erythrocytes maintained normal morphology. Co-culture with HUVECs and RAW264.7 was performed using different concentrations of DAT hydrogel, and live/dead fluorescence staining was performed at 1 d (Fig. S4A), 3 d (Fig. S4B), and 5 d (Fig. 4C) to observe the biocompatibility of DAT hydrogel. The results showed that most of the cells in the control and DAT hydrogel groups (4, 6, and 8 mg/ml) were alive (green), and only a few cells were dead (red). Thus, the DAT hydrogel showed ideal biocompatibility.

The proliferative effects of DAT hydrogels on HUVECs and RAW264.7 were detected by CCK-8 assay. On the first day, the differences in the optical density (OD) values of the groups were not statistically different. However, on day 3, the OD values of HUVECs increased with the concentration of DAT hydrogel, and the difference between 6 mg/ml DAT hydrogel and 8 mg/ml DAT hydrogel was not statistically significant. On day 5, the OD values of both RAW264.7 (Fig. 4D) and HUVECs (Fig. 4E) also increased with increasing concentration of DAT hydrogel, but 6 mg/ml DAT hydrogel had higher OD values than 8 mg/ml DAT hydrogel, and the difference was statistically significant. DAT hydrogel showed a solid ability to promote the growth of HUVECs and RAW264.7. The biocompatibility of DAT hydrogel was also further demonstrated. Different concentrations of DAT hydrogels had different abilities to promote cell proliferation, which provided a reference for selecting the appropriate proportion of hydrogels.

### In vitro angiogenesis-promoting function of DAT hydrogels

**Fig. 5.**
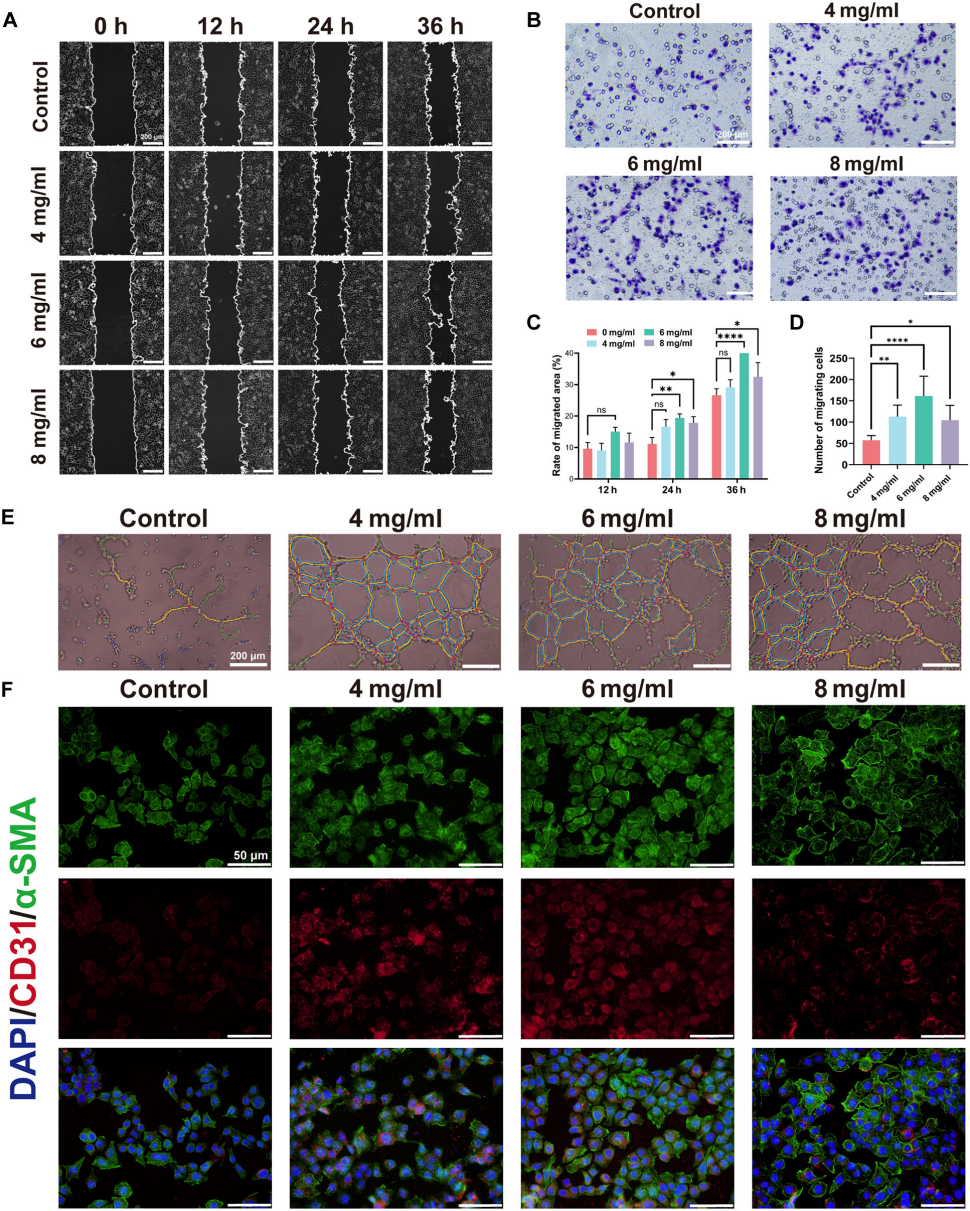
(A and C) Results of HUVEC migration ability assessed by wound healing assay (scale bar = 200 μm). (B) Determination of the invasive capacity of HUVECs by Transwell assay (scale bar = 200 μm). (D) Number of invasive cells in the Transwell assay. (E) Tube formation assay of HUVECs (scale bar = 200 μm). (F) Red-labeled (CD31) and green-labeled (α-SMA) immunostaining showed that DAT hydrogel-treated HUVECs exhibited enhanced angiogenic properties (scale bar = 50 μm). Statistical difference expression: ns, *P* > 0.05; *P < 0.05; **P < 0.01; ***P < 0.001; ****P < 0.0001; analyses were performed using ANOVA, *n* = 3.

Neovascularization plays an essential role in tissue healing [37]. A high-glucose environment restricts the induction of angiogenic factors, leaving angiogenic factors imbalanced with vasopressor factors, leading to impaired neoangiogenesis and vascular dysfunction [38,39]. Therefore, it is important to promote DM wound neovascularization. To investigate the ability of DAT hydrogel to promote vascular migration, we performed wound healing experiments and Transwell migration experiments. As shown in Fig. 5A and C, the wound healing area in the DAT hydrogel group was significantly larger than that in the control group, with the largest healing area in the 6 mg/ml group. Transwell migration experiments also led to the same conclusion: that adding DAT hydrogel promoted the migration of HUVECs (Fig. 5B and D). This reflects the fact that DAT hydrogels can significantly promote HUVEC migration, with the best promotion at 6 mg/ml.

In addition, we performed tube formation assay to test angiogenic capacity. The results showed that the DAT hydrogel group had more powerful angiogenesis than the control group (Fig. 5E). In addition, ImageJ analysis showed that the DAT hydrogel group had a more significant number of master junctions, nodes, and total segment length (Fig. S5).

We typically use CD31 protein expressions to characterize the ability to promote angiogenesis and, concurrently, α-SMA protein expression to characterize the ability to promote vessel maturation (Fig. 5F). In immunofluorescence against HUVECs, adding DAT hydrogel promotes the expression of CD31 and α-SMA proteins (Fig. S6). Together, these results confirm the positive effect of DAT hydrogels on the angiogenic capacity of HUVECs in vitro.

### In vitro anti-inflammatory and antioxidant effects of DAT hydrogel

**Fig. 6.**
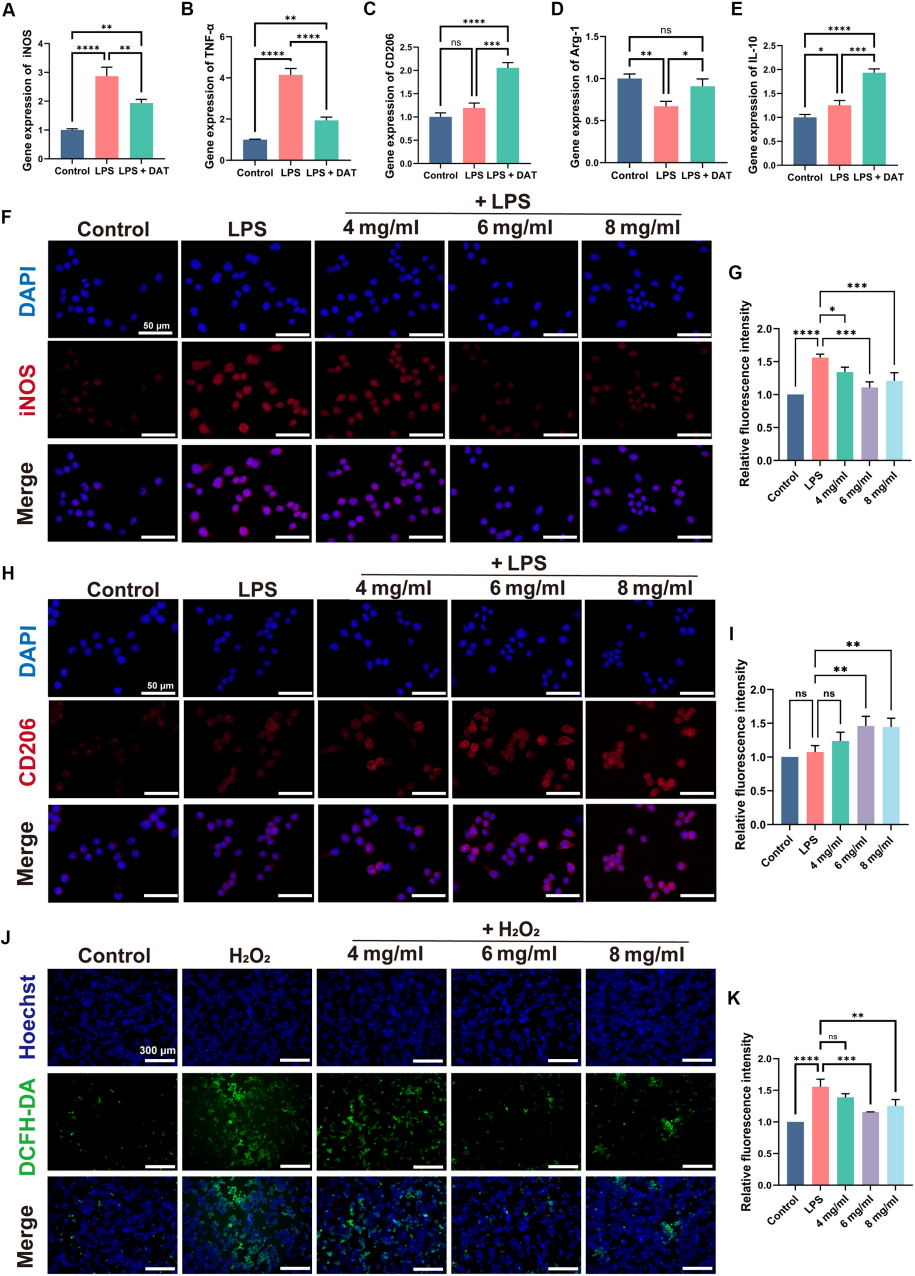
(A to E) Real-time reverse transcription polymerase chain reaction (RT-qPCR) results of the messenger RNA (mRNA) expression of anti-inflammatory factors (CD206, IL-10, and Arg-1) and inflammatory factors (iNOS and TNF-α). (F) Immunofluorescence of DAT hydrogels against iNOS expression (scale bar = 50 μm). (H) Immunofluorescence of CD206 expression by DAT hydrogel (scale bar = 50 μm). (G and I) Fluorescence intensity was quantified using ImageJ. (J and K) Antioxidant effect of DAT hydrogel on hydrogen peroxide-induced oxidative environment (scale bar = 300 μm). Statistical difference expression: ns, *P* > 0.05; *P < 0.05; **P < 0.01; ***P < 0.001; ****P < 0.0001; analyses were performed using ANOVA, *n* = 3. LPS, lipopolysaccharide; DCFH-DA, dichlorodihydrofluorescein diacetate.

The effect of a DM environment on wound healing is not only due to impaired neovascularization. It also promotes macrophage switching to an inflammatory phenotype (M1) [4,40] and increases local accumulation of ROS [41,42]. To verify whether DAT hydrogel can promote macrophage transformation from an M1 to an M2 type, we designed to use LPS to induce macrophage conversion to the M1 type while making DAT hydrogel to treat this conversion. We validate the effect of DAT hydrogel on the macrophage RAW 264.7 phenotype and anti-inflammatory effects using LPS as a negative control. RT-qPCR was performed to show the relative gene expression of pro-inflammatory and anti-inflammatory genes. The results showed that the expression of inflammatory genes (*iNOS* and *TNF-α*) was significantly increased in LPS-treated macrophages (Fig. 6A and B), and the expression of the inflammatory genes was decreased considerably after DAT hydrogel treatment. The expression of anti-inflammatory genes (*Arg-1*, *Cd206*, and *Il10*) was also significantly higher after DAT hydrogel treatment compared that of the LPS group (Fig. 6C to E). At the same time, we performed immunofluorescence staining of macrophages for *Cd206* (M2 type) and *iNOS* (M1 type). The results showed that LPS significantly increased *iNOS* expression, while DAT hydrogel treatment significantly decreased this expression (Fig. 6F and G). Meanwhile, both LPS and DAT hydrogel increased *Cd206* expression, but that of the DAT hydrogel was significantly higher than that of the LPS group (Fig. 6H and I). These experiments showed that DAT hydrogel promotes the polarization of pro-inflammatory M1 macrophages into pro-healing M2 macrophages and reduces inflammation.

In order to evaluate the antioxidant properties of DAT hydrogels, DCFH-DA was employed as a H_2_O_2_ probe, while Hoechst was utilized as a cell nuclear stain. The findings demonstrated that the DAT hydrogel was efficacious in reducing the ROS level induced by H_2_O_2_ in a dose-dependent manner. Meanwhile, there was no significant difference between different concentrations of DAT hydrogels in reducing ROS levels (Fig. 6J and K). These results suggest that DAT hydrogel has the ability to scavenge ROS and antioxidant potential. In addition, we performed live/dead staining after 6 h of H_2_O_2_ induction, and the results showed that cell death increased after H_2_O_2_ induction. However, dead cells decreased after DAT hydrogel treatment (Fig. S7). In conclusion, these results suggest that DAT hydrogel effectively modulates macrophage polarization toward an anti-inflammatory M2 phenotype and promotes ROS clearance.

### DAT hydrogel promotes soft-tissue healing in the palate of DM rats

An essential consideration in oral mucosa wound healing is the maintenance of therapeutic agents in a constantly wet and mechanically active environment. To address this, we employed a submucosal injection strategy to localize DAT hydrogel beneath the mucosal layer, thereby physically isolating it from direct salivary exposure and mechanical abrasion. Before verifying the healing ability of DAT hydrogel on DM oral wounds, we performed subcutaneous injections on the back of rats. The in vivo biocompatibility of DAT hydrogel was observed by HE staining of subcutaneous and critical organs. As shown in Fig. S8, no obvious structural abnormalities or local inflammatory reactions were observed in rats’ subcutaneous injection sites (the injection sites are indicated by arrows). Also, HE staining of key organs did not show obvious abnormalities (Fig. S9). These results indicate that DAT hydrogel has good biocompatibility and can be used for submucosal injections.

**Fig. 7.**
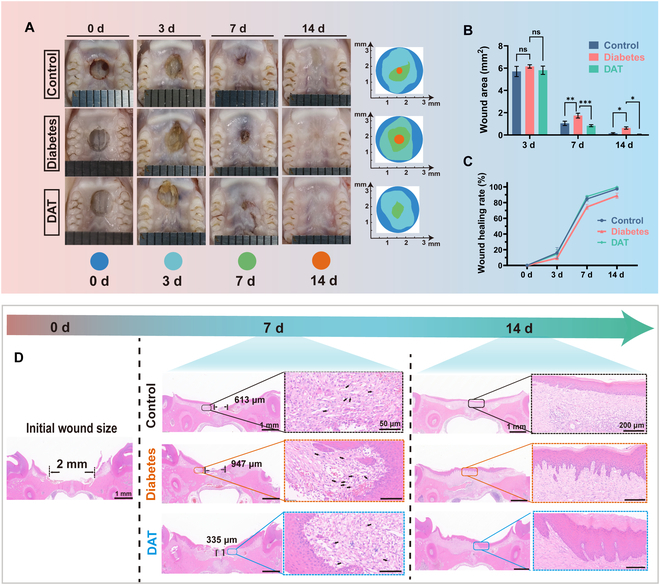
(A) Naked eye view of wound healing in DM rats at different times. (B) A plot of palatal wound area analysis was performed in DM rats at different time points. (C) Line graph of wound healing ratio over time. (D) HE-stained sections of palatal wounds of DM rats at different times. Black arrows point to inflammatory cells (scale bar = 1 mm, scale bar of enlarged diagrams = 50 μm [7 d] and 200 μm [14 d]). Statistical difference expression: ns, *P* > 0.05; *P < 0.05; **P < 0.01; ***P < 0.001; analyses were performed using ANOVA, *n* = 3.

The DAT hydrogel showed good biocompatibility in both vivo and in vitro experiments. Subsequently, we used a 2-mm-diameter perforator to create a homogeneous soft-tissue defect in the palate of DM SD rats, which reached right up to the bone surface at a depth of approximately 0.5 mm (Fig. S10). Subsequently, wound healing was promoted by intramucosal injection of DAT hydrogel, and the wound area was observed on days 3, 7, and 14 (Fig. 7A). We found that on day 3, there was little difference in healing area among the 3 groups. By day 7, the healing area of the DAT hydrogel group was significantly larger than that of the DM group, while there was no significant difference from the control group. Although the macroscopic wound closure rate of diabetic mice appeared to be comparable to that of the control group on day 7, quantitative image analysis and histopathologic examination showed significant differences. Finally, on day 14, the wounds were healed in the DAT hydrogel group and the control group but remained unhealed in the DM group (Fig. 7B and C). In conclusion, in terms of wound area, DM rats showed slower wound healing than normal rats, while DAT hydrogel promoted wound healing.

To further evaluate the healing process of the wound, we performed HE and Masson trichrome staining of the periphery of the wound. As shown in Fig. 7D, on day 0, a 2-mm-diameter flush soft-tissue defect in the palate’s center reached right down to the bone surface. The wound healed gradually over time, with the DM group healing the slowest and the DAT hydrogel group healing the fastest. At the same time, we could find typical diabetic healing (increased inflammatory cell infiltration, disturbed collagen alignment, and delayed epithelialization) in the histological sections. The wound healed gradually over time. The DM group healed the slowest, while the DAT hydrogel group healed the fastest. Peri-wound HE staining from day 7 (Fig. 7D) showed more inflammatory cells in the DM group, while inflammatory cells were significantly reduced after DAT hydrogel treatment. Meanwhile, peri-wound HE staining on day 14 (Fig. 7D) showed that the epithelial pegs were elongated and irregular in the DM group. In contrast, the epithelial layer was widened, and the pegs were not apparent after DAT hydrogel treatment. Meanwhile, immunohistochemical staining (TNF-α and IL-1β) of rats on day 7 showed that DM had more positive cells, while the number of positive cells decreased after DAT treatment (Fig. S11). The results of HE and immunohistochemistry (TNF-α and IL-1β) showed that DM exacerbated the inflammatory response to oral trauma, whereas the addition of DAT attenuated the inflammatory response.

The oral mucosa lacks a fat layer compared to the skin and requires more connective tissue padding [43]. For DM wounds, the high-glucose environment leads to suboptimal filling of connective tissue by reducing collagen expression [44,45]. Therefore, the deposition of collagen fibers is an essential signal for assessing the wound healing process. Masson staining examined collagen deposition within the tissue surrounding the incision. The Masson staining results shown in Fig. S12 showed that on day 7, the collagen fiber multiplication around the peri-incision margins of the 3 groups did not differ much and were all disordered. However, on day 14, the collagen fiber multiplication area in the DAT hydrogel group was significantly higher in the control and DM groups, and the arrangement was more regular. The ordered accumulation of collagen fibers in the DAT hydrogel group demonstrated that the treatment facilitated the healing of wounds. Meanwhile, in the present study, daily administration of DAT hydrogel was used for the preliminary validation of the effectiveness of DAT hydrogel. However, further studies are needed to determine whether a shorter dosing frequency can bring about the same therapeutic effect.

### DAT hydrogel promotes angiogenesis and anti-inflammation in the intraoral wounds of DM rats

**Fig. 8.**
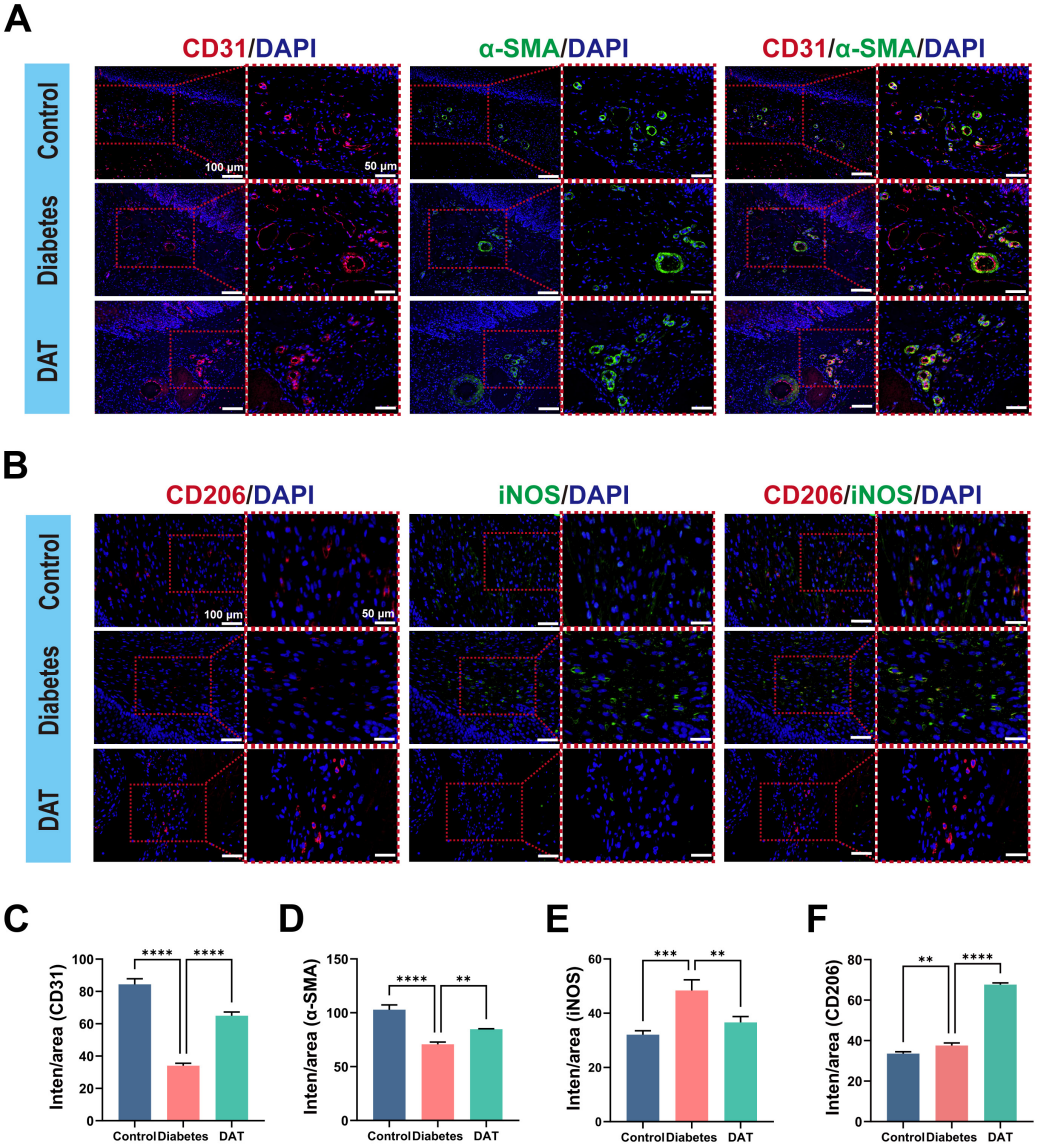
(A) Immunofluorescence staining of angiogenesis in the oral wounds of DM rats on the seventh day of healing (scale bar = 100 μm). (B) Anti-inflammatory immunofluorescence staining of the oral wounds of DM rats on the seventh day of healing (scale bar = 100 μm). (C to F) Fluorescence intensity was quantified using ImageJ. Statistical difference expression: **P < 0.01; ***P < 0.001; ****P < 0.0001; analyses were performed using ANOVA, *n* = 3. Inten, intensity.

As mentioned earlier, DM can lead to impaired vascularization of the trauma. Therefore, we used CD31 and α-SMA to characterize peri-wound angiogenesis and vessel maturation (Fig. 8A). The fluorescence intensity of anti-CD31 and α-SMA antibodies, as well as the number of blood vessel formations, was significantly greater in the DAT hydrogel group than in the DM group (Fig. 8C and D). This surface DM causes a reduction in peri-wound blood vessel formation and maturation, which DAT hydrogel promotes.

In addition, the high-glycemic environment caused by DM brings about excessive inflammation by promoting macrophage polarization toward the M1 type [5,46], and excessive inflammation often leads to slow tissue healing [47]. The effect of the DAT hydrogel on macrophage phenotypic transformation was investigated using immunofluorescence staining, which enabled the detection of macrophages. iNOS and CD206 were chosen as surface markers for M1-type and M2-type macrophages, respectively (Fig. 8B). Compared to the control group, there was a significant increase in iNOS^+^ macrophages in the DM group (Fig. 8E and F). In contrast, DAT hydrogel significantly decreased the percentage of INOS^+^ macrophages and increased the percentage of CD206-positive macrophages. These findings suggest that DAT hydrogel has anti-inflammatory effects at the wound site. The findings illustrate that the DAT hydrogel is an efficacious agent for the reduction of inflammatory processes at the site of injury and the promotion of new blood vessel formation.

### Preliminary exploration of the healing mechanism of DAT hydrogels

**Fig. 9.**
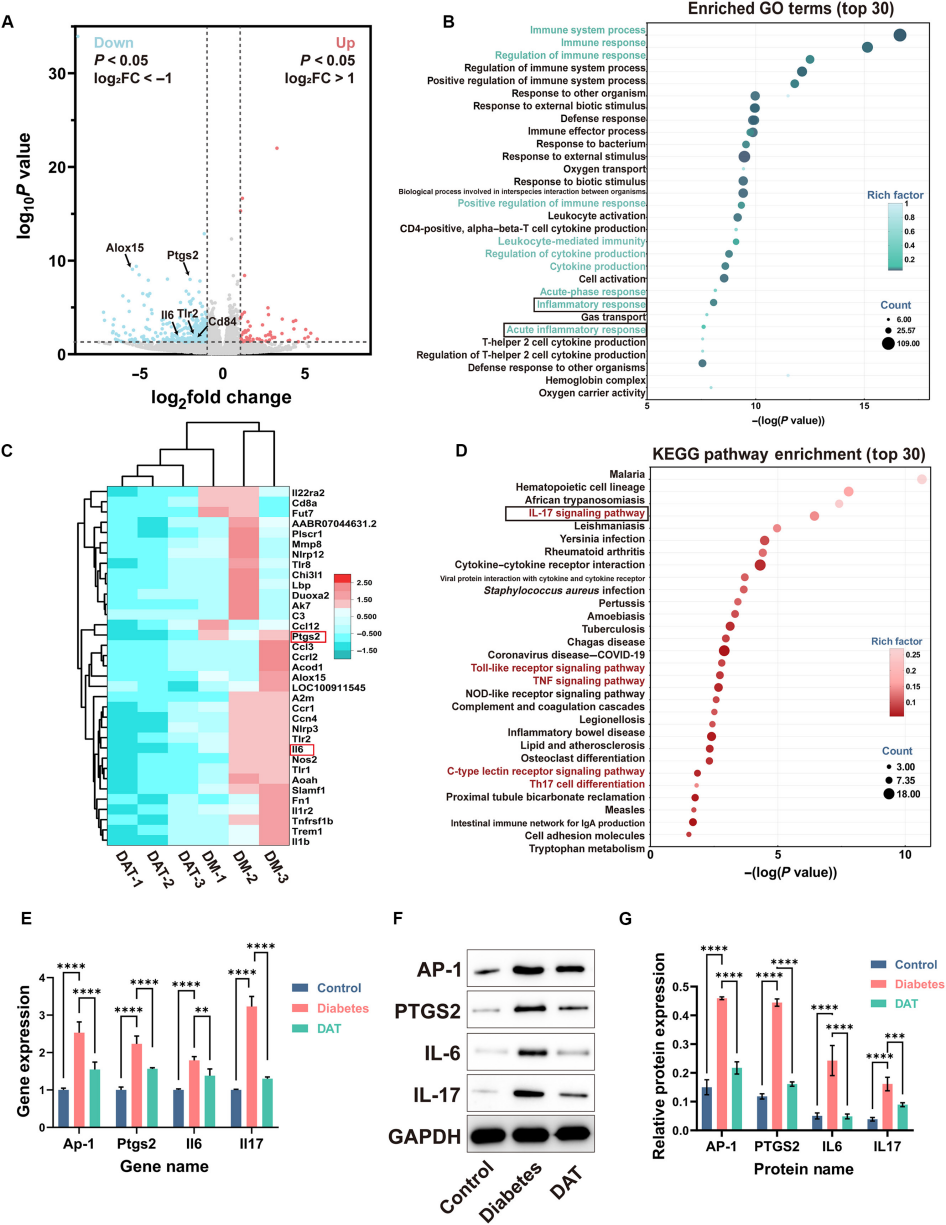
(A) Volcano plots with |log fold change| > 1 and *P* values < 0.05 as thresholds. (B) Differential gene heat map. (C) The 30 most enriched entries in the GO analysis. (D) Heat map of inflammation-related differential genes. (E) The 30 most enriched pathways in KEGG pathway analysis. (F) Protein expression of IL-17 pathway-related proteins (AP-1, PTGS2, IL-6, and IL-17). (G) Grayscale semiquantitative analysis of related protein bands. Statistical difference expression: ns, *P* > 0.05; *P < 0.05; **P < 0.01; ***P < 0.001; ****P < 0.0001; analyses were performed using ANOVA, *n* = 3. FC, fold change.

To further understand the mechanism by which DAT hydrogel promotes DM intraoral wound healing, we performed RNA-seq on the soft tissues of the palate of rats on the seventh day when the difference in healing was the greatest (Table S4). As shown in the volcano plot in Fig. 9A, when the threshold for significance was set at log fold change > 1 and *P* value < 0.05, we identified 377 down-regulated genes and 66 up-regulated genes. Differential genes were represented by Fig. 9A and the heat map in Fig. S13, and many pro-inflammatory genes were down-regulated in the DAT hydrogel set: *Alox15*, *Ptgs2*, *Il6*, *Tlr2*, *Cd84*, and *Tlr1*. GO enrichment analysis was subsequently performed and categorized into 3 main groups: biological process, cellular component, and molecular function. We collated the 30 most relevant entries in the 3 main categories (Fig. 9B). The bubble diagram shows that the differential expression between the DM group and the DAT hydrogel group was closely related to inflammatory biological processes such as immune system process, immune response, inflammatory response, and leukocyte-mediated immunity. Meanwhile, we analyzed inflammation-related differential genes and showed that DAT hydrogel down-regulated the vast majority of inflammatory genes (Fig. 9C). The pathways were analyzed in KEGG. The 30 most relevant entries were compiled (Fig. 9D). It was found that the IL-17 signaling pathway, TNF signaling pathway, and C-type lectin receptor signaling pathway among the signaling pathways might be closely related to the promotion of tissue growth through anti-inflammation by DAT hydrogel. The IL-17 signaling pathway is one of the most critical anti-inflammatory signaling pathways in various DM inflammatory diseases [48]. At the same time, we found that several of the above down-regulated inflammatory genes (*Il6* and *Ptgs2*) were associated with the IL-17 signaling pathway.

To investigate whether the IL-17 signaling pathway is a key target of DAT hydrogel for treating DM oral soft-tissue wounds, we used RT-qPCR and Western blotting to verify the underlying mechanism. As shown in Fig. 9F, the expression of key gene and protein (IL-17, AP-1, IL-6, and PTGS2) levels were significantly up-regulated in DM rats compared to normal rats (Fig. 9E). However, this trend was partially reversed after DAT hydrogel treatment (Fig. 9F and G), suggesting that the DAT hydrogel could attenuate inflammation and promote DM oral soft-tissue healing by modulating the IL-17 pathway.

## Conclusion

Promoting oral wound healing in DM patients remains a major challenge. In this study, we innovatively hypothesized that DAT has relevant proteins to promote DM wound healing by shotgun proteomic analysis. Subsequently, an injectable, temperature-sensitive DAT hydrogel was successfully synthesized to adapt to the moist, friction-rich oral environment, and its mechanism of action was explored. In subsequent studies, we found that this hydrogel could promote angiogenesis by promoting HUVEC proliferation, migration, and expression of CD31 and α-SMA. It also promoted the conversion of macrophages from a pro-inflammatory phenotype (M1) to an anti-inflammatory phenotype (M2). The hydrogel attenuated the exacerbation of DM-induced inflammation through the IL-17 pathway. It promoted the healing of oral wounds in DM rats, as preliminarily confirmed by protein expression characterization through transcriptomic analysis. In conclusion, we found and validated that DAT hydrogel could be used as a new strategy to promote oral wound healing in DM through a multi-omic approach. Meanwhile, the present study also provides a new approach to an in-depth analysis of the potential mechanism of biomaterials.

## Data Availability

The analyzed datasets generated during this study are available from the corresponding author on reasonable request.
