# Supplementary material for: The Extracellular Matrix Promotes Diabetic Oral Wound Healing by Modulating the Microenvironment
Source: Biomater Res. 2025 Mar 19;29:0169. doi: 10.34133/bmr.0169 (PMC11922533; doi:10.34133/bmr.0169)
Supplement: Supplementary 1 — Tables S1 to S4 Figs. S1 to S13 [file bmr.0169.f1.zip › Supplemental Information.docx]

**Supplemental Information**

**Oral environment-adapted decellularized extracellular matrix promotes tissue healing by Modulating the microenvironment of oral diabetic wounds**

Zhongke Wang ^a b c^, Li Wang ^a b c^, Sihan Wang ^a b c^, Hongmei Chen ^a b c^, Danni Wang ^a b c^, Aodi Li ^a b c^, Ying, Huang ^a b c^, Yifan Pu ^a b c^, Xinlei Xiong ^a b c^, Xiangrui Lui ^a b c^, Yuwen Huang ^a b c^, Ling Guo ^a b c^

^a^ Department of Prosthodontics, The Affiliated Stomatological Hospital of Southwest Medical University, Luzhou, 646000, China.

^b^ School of Stomatology, Southwest Medical University, Luzhou, Sichuan, China.

^c^ Luzhou Key Laboratory of Oral & Maxillofacial Reconstruction and Regeneration, Luzhou, 646000, China.

**Corresponding Author:** Ling Guo, The Affiliated Stomatology Hospital of Southwest Medical University, 2 Jiang Yang South Road, Lu Zhou, Sichuan 646000, P.R. China. Email: glsmiling@swmu.edu.cn

Table S1

Primer sequences used for RT-qPCR analysis.

| Gene | Primer sequence |
| --- | --- |
| *β-actin* | F: CCCATCTACGAGGGCTAT |
|  | R: TGTCACGCACGATTTCC |
| *Inos* | F: GCTAATGCGAAAGGTCA |
|  | R: TGGTGTTGAAGGCGTAG |
| *Tnf-α* | F: GCCTCTTCTCATTCCTGCTTG |
|  | R: CTGATGAGAGGGAGGCCATT |
| *Cd206* | F: GGTGGCTTATGGGATGTTT |
|  | R: GGGTTCAGGAGTTGTTGTG |
| *Il-10* | F: ACCTGGTAGAAGTGATGCC |
|  | R: GACACCTTGGTCTTGGAG |
| *Arg-1* | F: GCTGGTCTGCTGGAAAAACT |
|  | R: CCACCCAAATGACACATAGGT |

Table S2

Primer sequences used for RT-qPCR analysis.

| Gene | Primer sequence |
| --- | --- |
| *Gapdh* | F: CAAGTTCAACGGCACAG |
|  | R: CCAGTAGACTCCACGACAT |
| *Ap-1* | F: ATGGGCACATCACCACTACAC |
|  | R: GCAGCGTATTCTGGCTATGC |
| *Ptgs2* | F: CTTCGGGAGCACAACAGAG |
|  | R: GCGGATGCCAGTGATAGAG |
| *Il6* | F: TGGAGTTCCGTTTCTACCTGG |
|  | R: GGATGGTCTTGGTCCTTAGCC |
| *Il-17* | F: GCCTGATGCTGTTGCTGC |
|  | R: AAGTGGAACGGTTGAGGTAGTC |


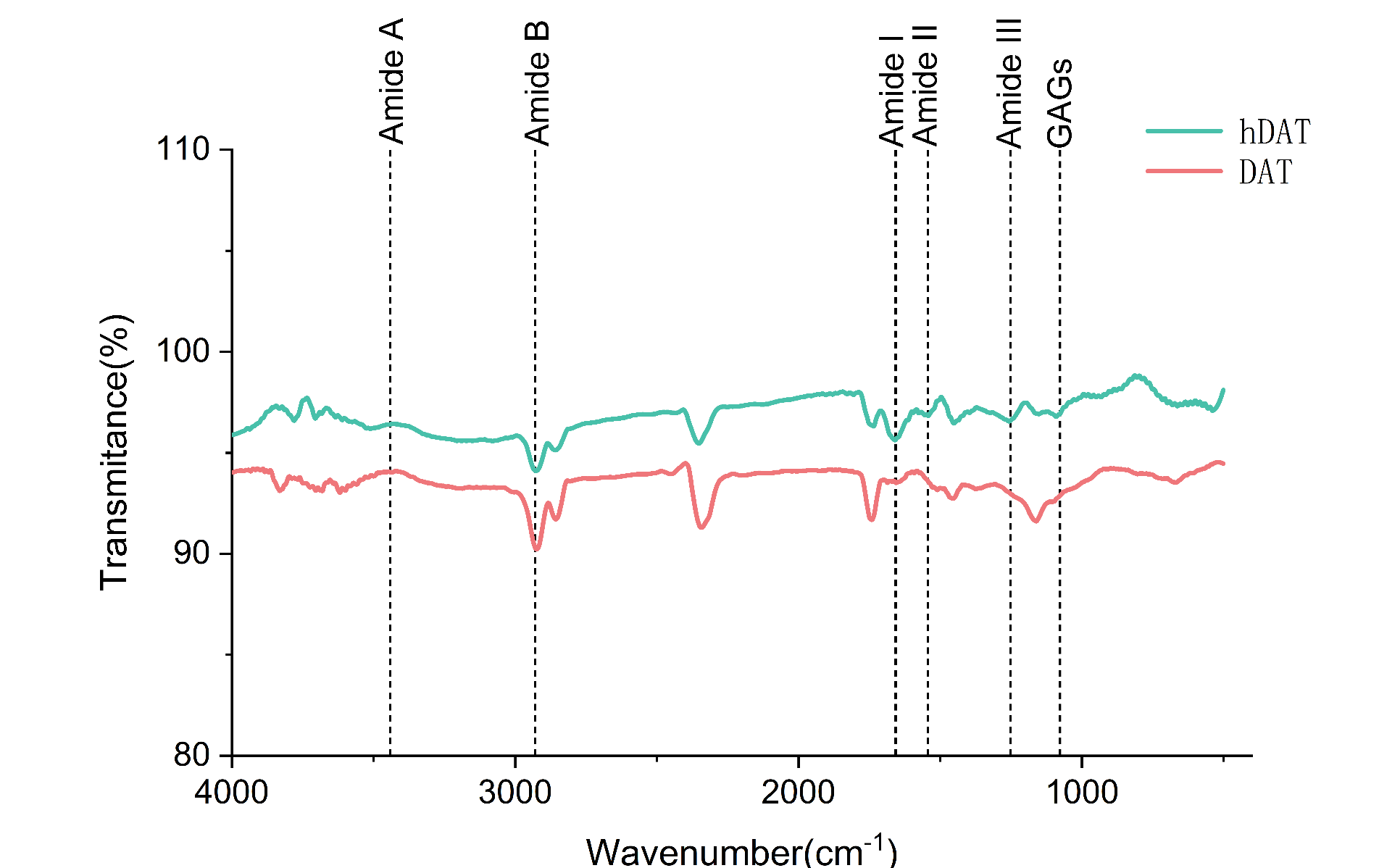


Fig. S1. Fourier infrared spectra of DAT and DAT hydrogels.


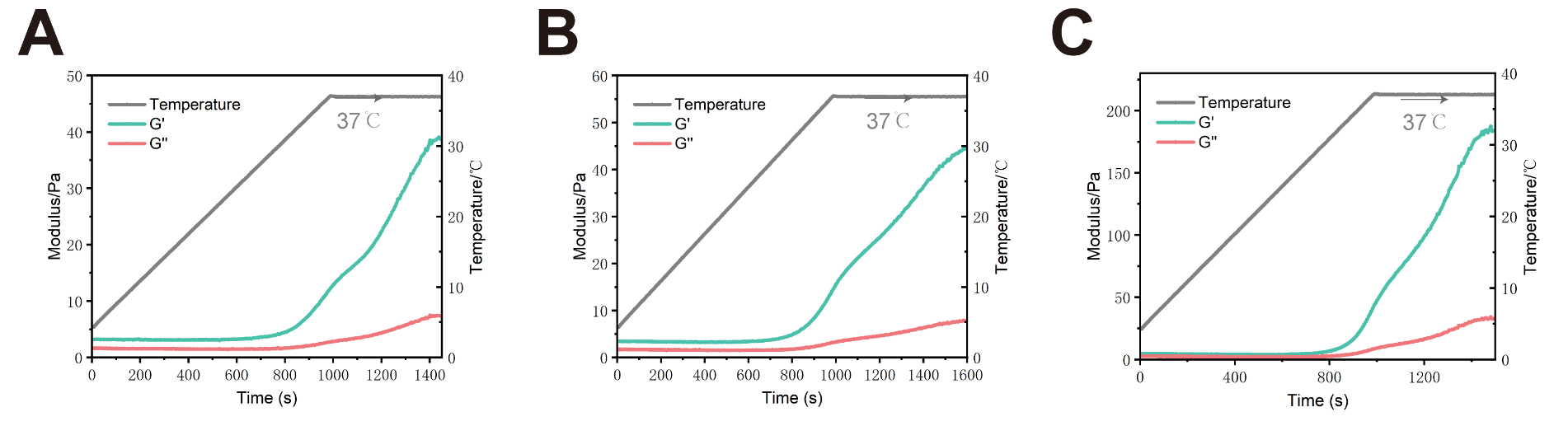


Fig. S2. Rheological analysis (energy storage modulus G', loss modulus G") of DAT hydrogels at different concentrations (4mg/ml (A), 6mg/ml (B),8mg/ml (C))


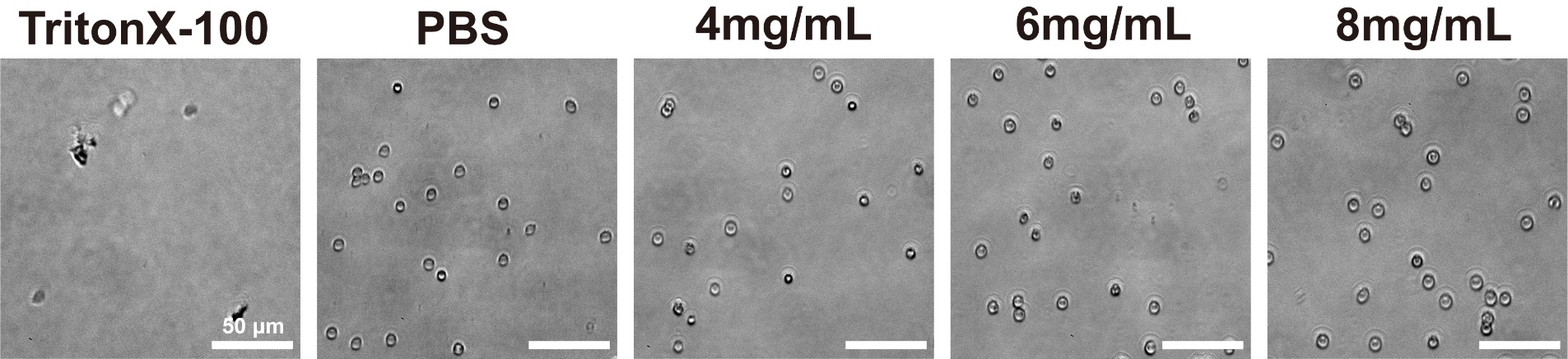


Fig. S3. Microscopic view of hemolysis experiments (scale bar = 50 μm).


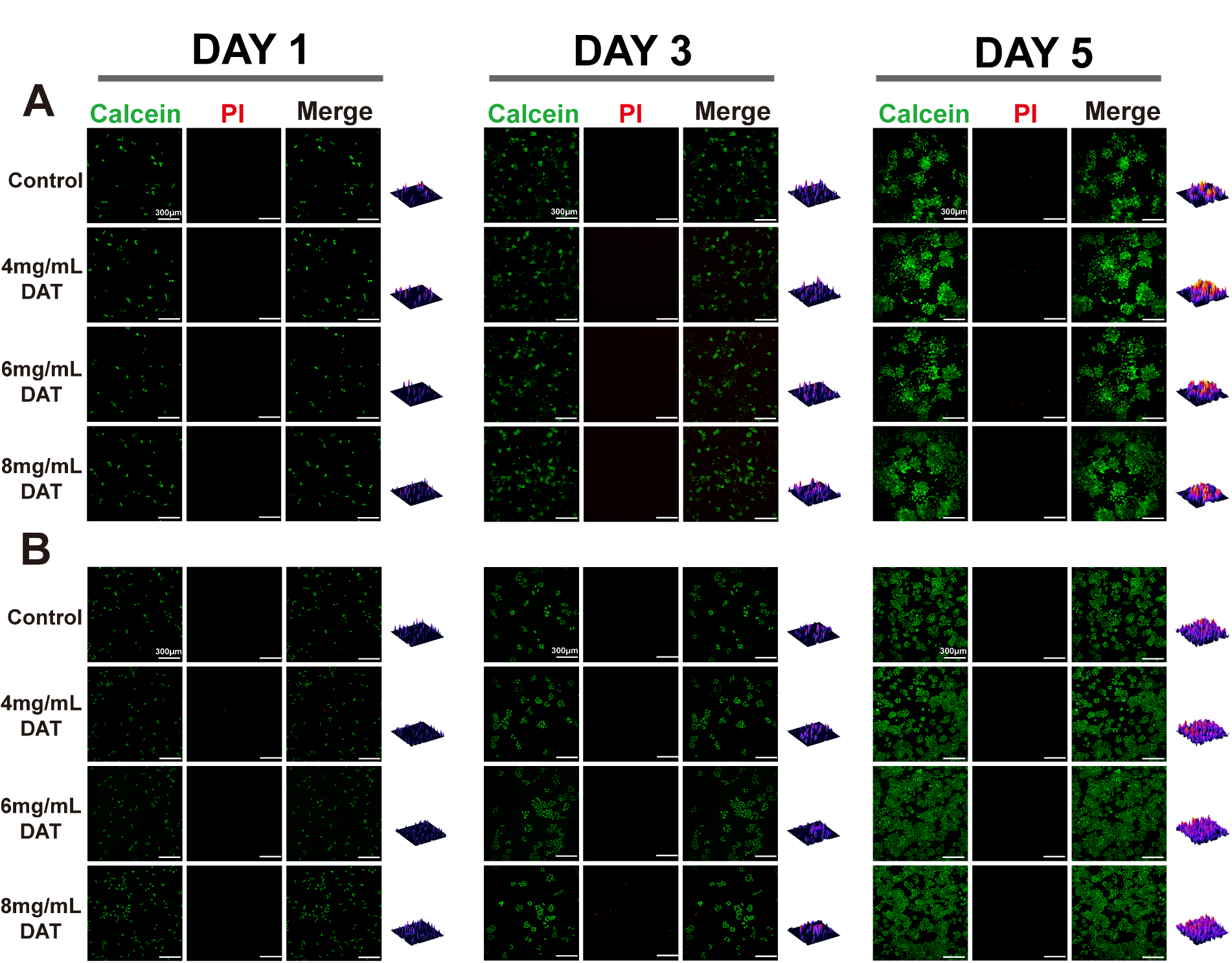


Fig. S4. Live/dead staining to assess the toxic effect of hydrogel (A: RAW267.4, B: HUVEC, scale bar = 300 μm).


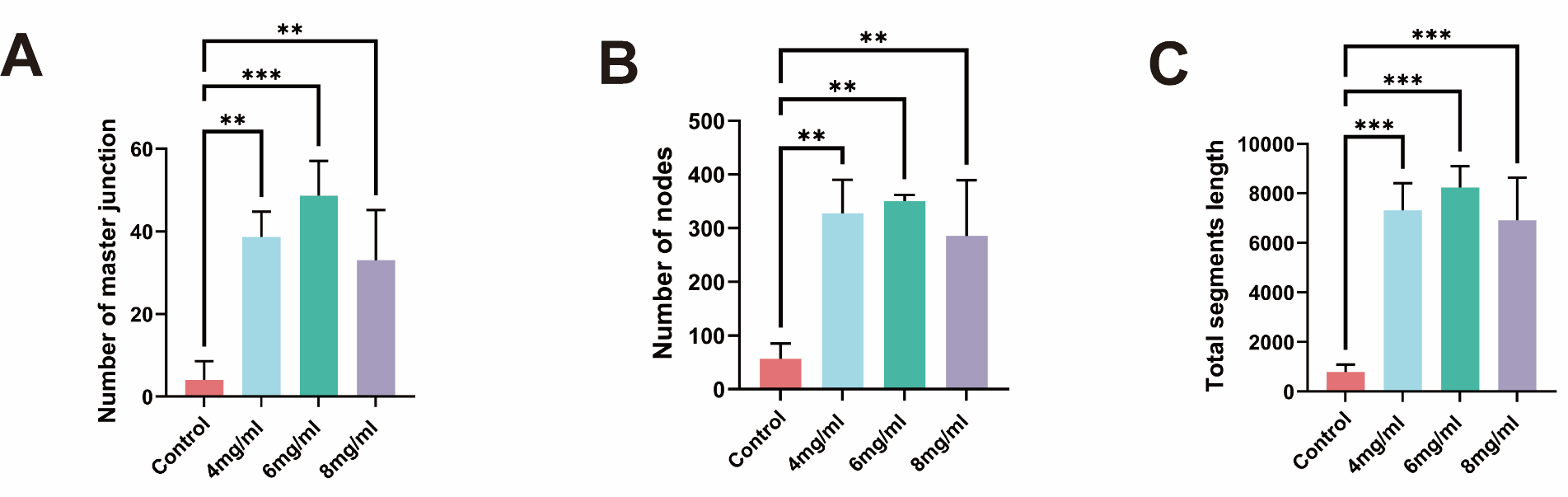


Fig. S5. Image J analysis showed that the DAT hydrogel group measured a more significant number of master junctions (A), number of nodes (B), and total segment length (C). ∗∗ *P* < 0.01 ∗∗∗, *P* < 0.001, n = 3.


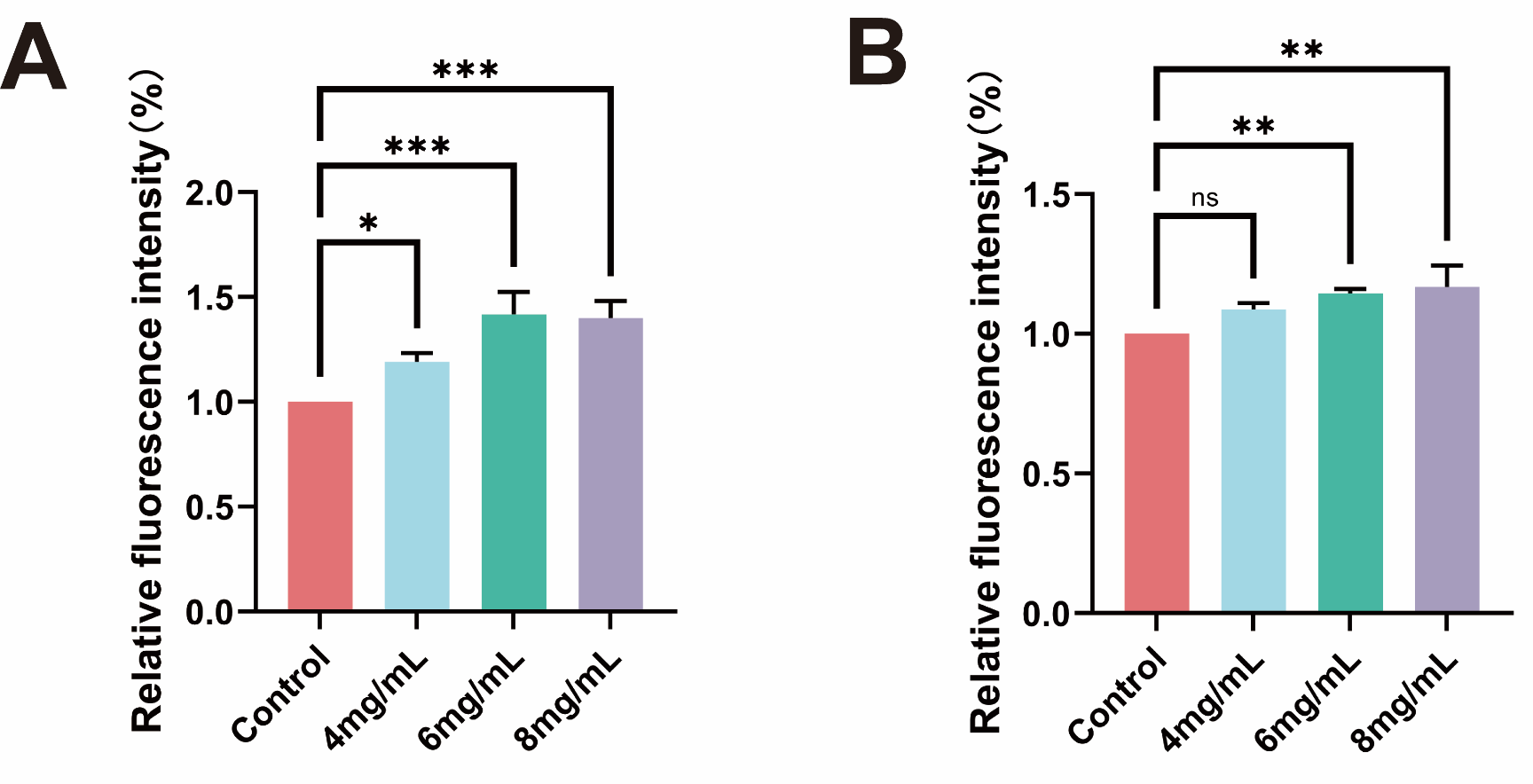


Fig. S6. Fluorescence intensity was quantified using Image J (A: CD31, B: α-SMA). ns *P* > 0.05, ∗ *P* < 0.05, ∗∗ *P* < 0.01 ∗∗∗, *P* < 0.001, n = 3.


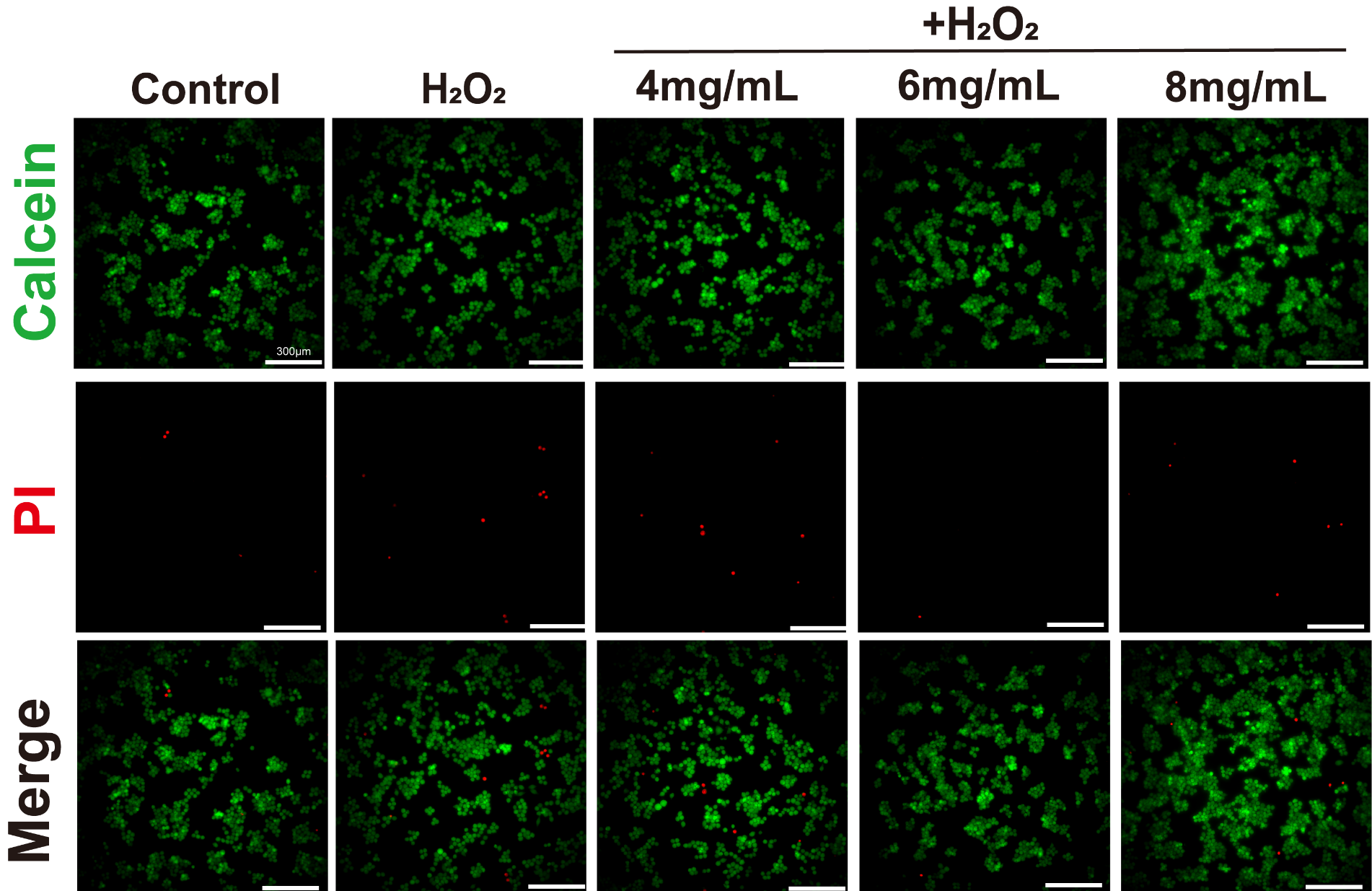


Fig. S7. Live-dead staining of cells in hydrogen peroxide environment by DAT hydrogel (Scale bar = 300 μm).


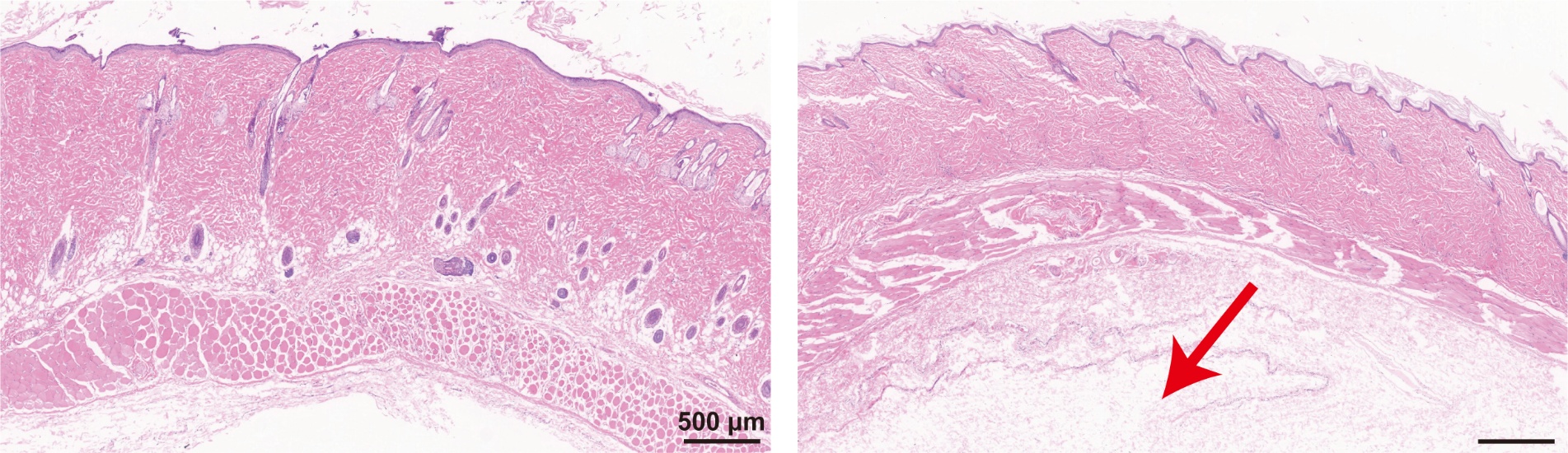


Fig. S8. HE staining of rat dorsal subcutaneous injection of DAT hydrogel with saline injection (Scale bar = 500 μm)

.


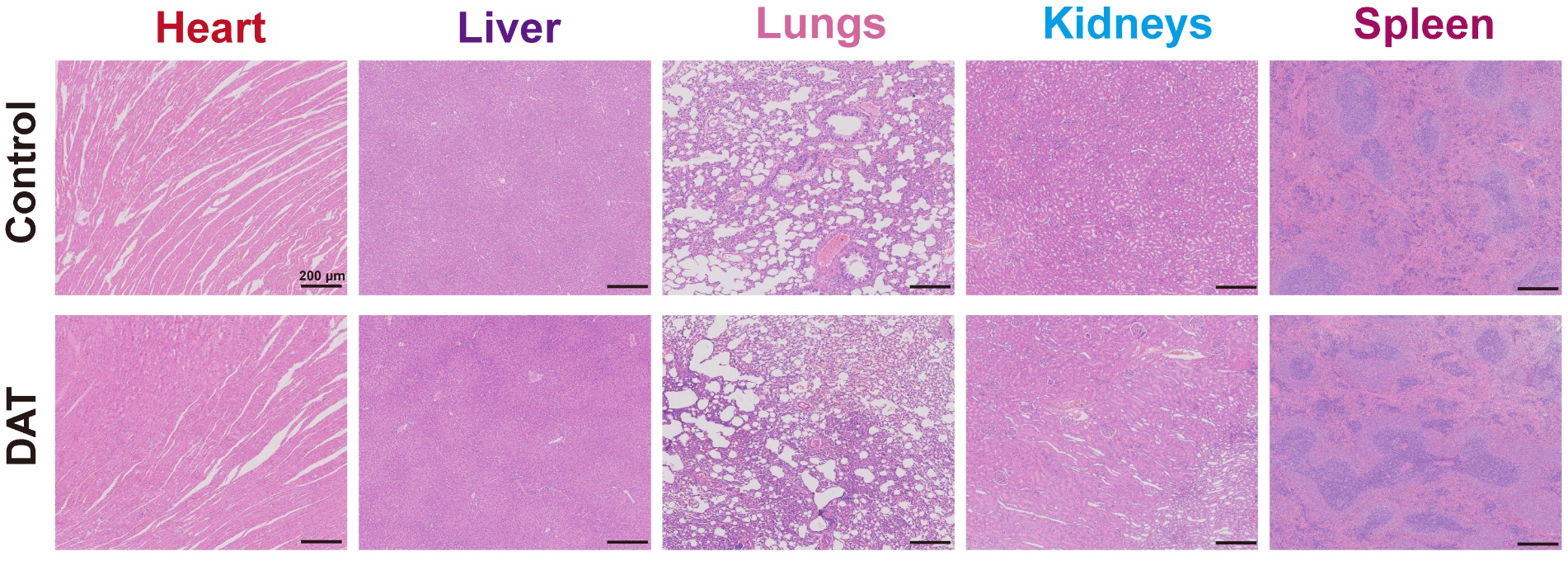


Fig. S9. HE staining of major organs after subcutaneous injection of DAT hydrogel in the back of rats (Scale bar = 200 μm)

.


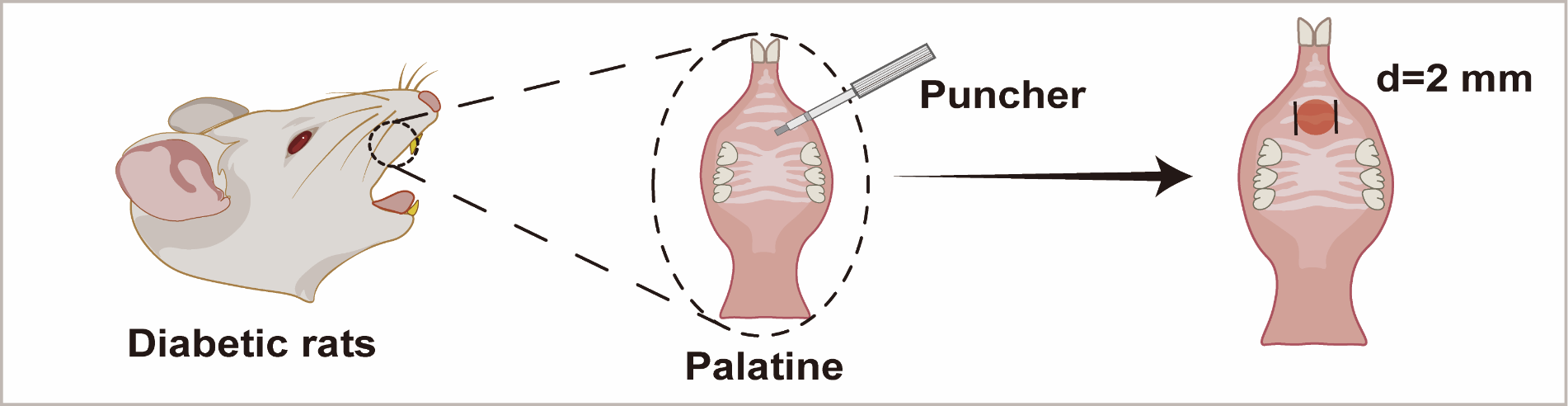
Fig. S10 DM rat animal modeling schematic diagram.


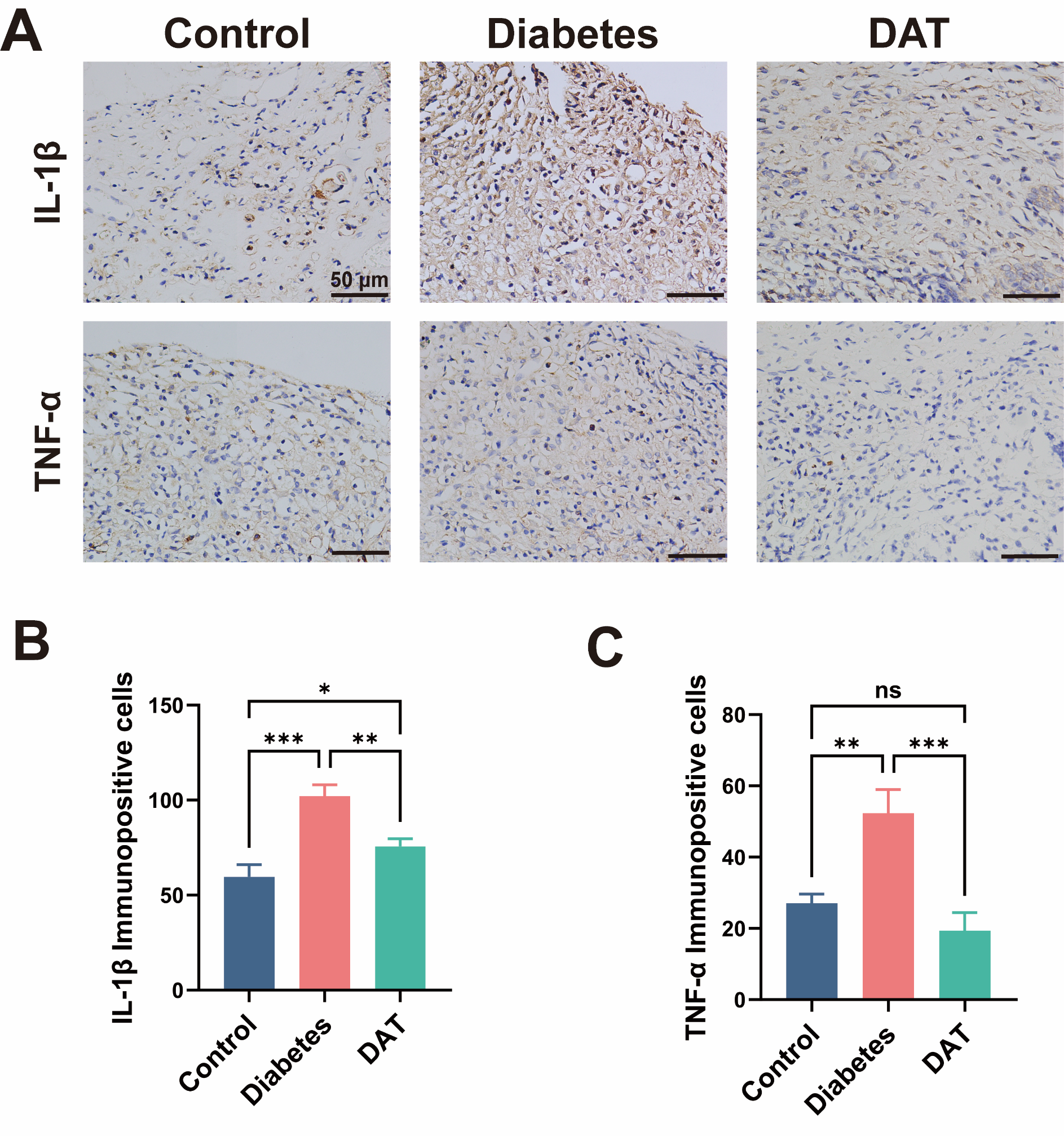


Fig. S11 (A) Immunohistochemical staining of rats on day 7 (TNF-α, IL-1β) (Scale bar = 500 μm, scale bar of enlarged diagrams = 50 μm) (B, C) Immunohistochemical positive cell count. Statistical difference expression: ns *P* > 0.05, ∗ *P* < 0.05, ∗∗ *P* < 0.01, ∗∗∗ *P* < 0.001, analyses were performed using ANOVA, n = 3.

.


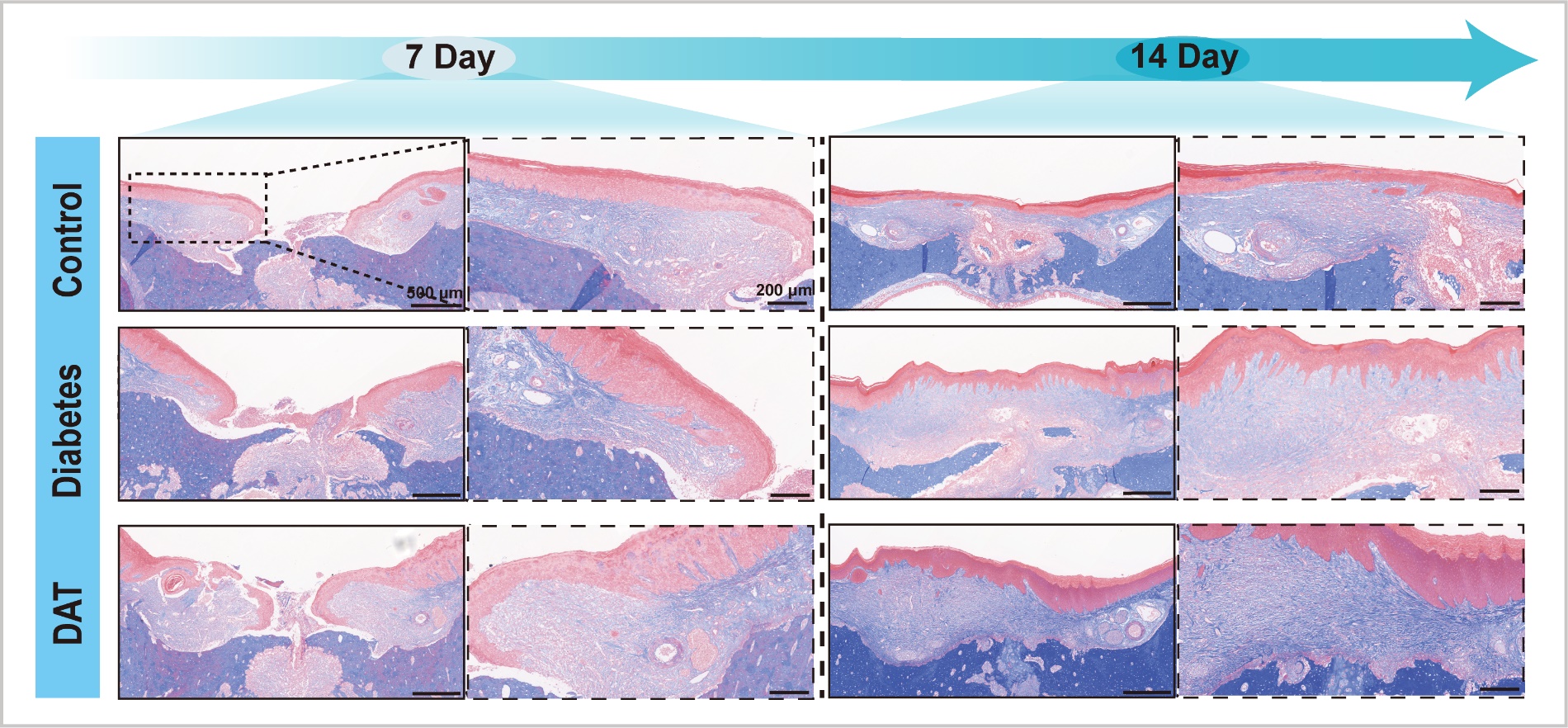


Fig. S12. MASSON-stained sections of palatal wounds of DM rats at different times (Scale bar = 500 μm, scale bar of enlarged diagrams = 200 μm)


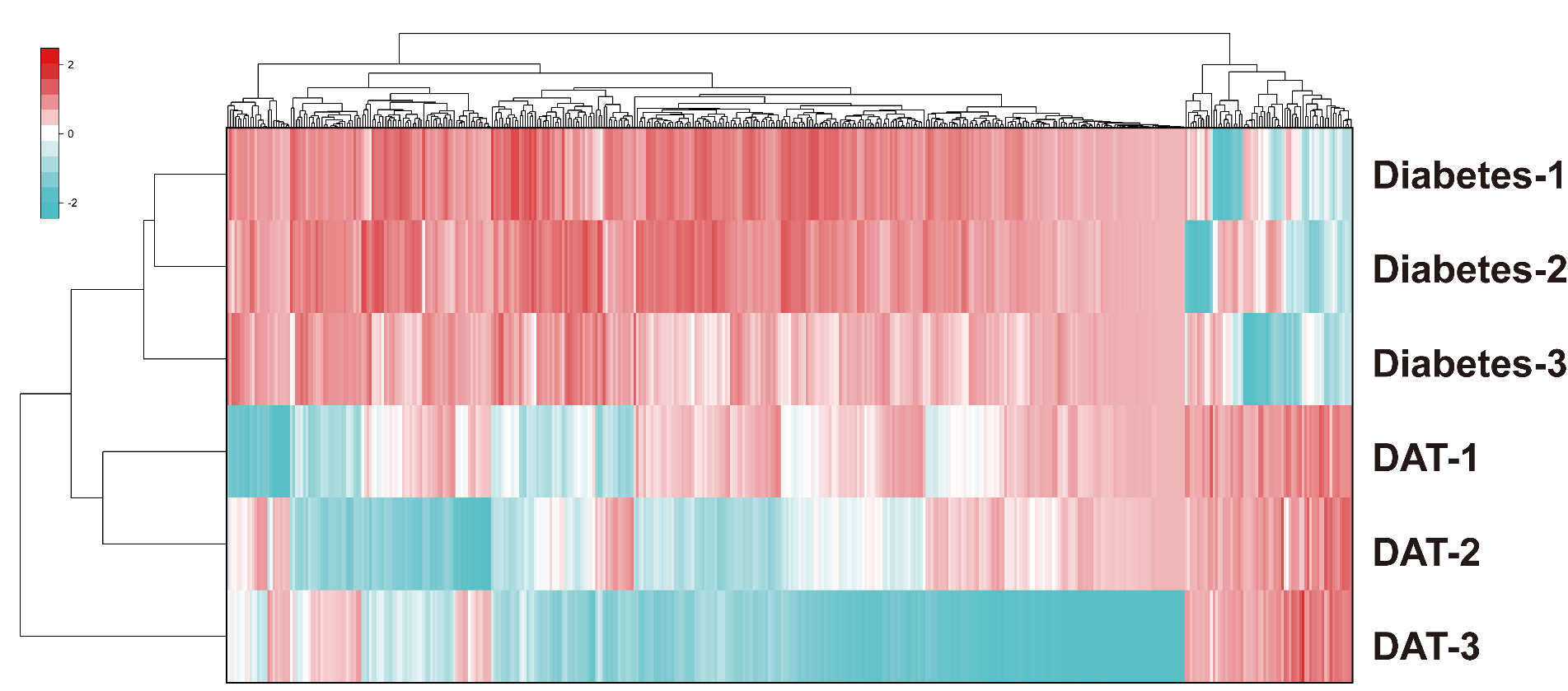


Fig. S13. Differential gene heat map.
